# A systems-based framework to computationally describe putative transcription factors and signaling pathways regulating glycan biosynthesis

**DOI:** 10.3762/bjoc.17.119

**Published:** 2021-07-22

**Authors:** Theodore Groth, Rudiyanto Gunawan, Sriram Neelamegham

**Affiliations:** 1 Chemical and Biological Engineering, University at Buffalo, State University of New York, Buffalo, NY 14260, USA; 2 Biomedical Engineering, University at Buffalo, State University of New York, Buffalo, NY 14260, USA; 3 Medicine, University at Buffalo, State University of New York, Buffalo, NY 14260, USA

**Keywords:** ChIP-Seq, glycoinformatics, glycosylation, TCGA transcription factor

## Abstract

Glycosylation is a common posttranslational modification, and glycan biosynthesis is regulated by a set of glycogenes. The role of transcription factors (TFs) in regulating the glycogenes and related glycosylation pathways is largely unknown. In this work, we performed data mining of TF–glycogene relationships from the Cistrome Cancer database (DB), which integrates chromatin immunoprecipitation sequencing (ChIP-Seq) and RNA-Seq data to constitute regulatory relationships. In total, we observed 22,654 potentially significant TF–glycogene relationships, which include interactions involving 526 unique TFs and 341 glycogenes that span 29 the Cancer Genome Atlas (TCGA) cancer types. Here, TF–glycogene interactions appeared in clusters or so-called communities, suggesting that changes in single TF expression during both health and disease may affect multiple carbohydrate structures. Upon applying the Fisher’s exact test along with glycogene pathway classification, we identified TFs that may specifically regulate the biosynthesis of individual glycan types. Integration with Reactome DB knowledge provided an avenue to relate cell-signaling pathways to TFs and cellular glycosylation state. Whereas analysis results are presented for all 29 cancer types, specific focus is placed on human luminal and basal breast cancer disease progression. Overall, the article presents a computational approach to describe TF–glycogene relationships, the starting point for experimental system-wide validation.

## Introduction

The glycan signatures of cells and tissue are controlled by the expression pattern of 300–350 glycosylating-related genes that are together termed glycogenes [1–2]. These glycogenes include the glycosyltransferases, glycosidases, sulfotransferases, transporters, etc. The expression of these glycogenes is in turn driven by the action of a class of proteins called transcription factors (TFs). These TFs regulate gene expression by binding proximal to the promoter regions of genes, facilitating the binding of RNA polymerases. They may homotropically or heterotropically associate with additional TFs in order to directly or indirectly control messenger RNA (mRNA) expression. Among the TFs, some “pioneer factors” can pervasively regulate gene regulatory circuits and access chromatin despite it being in a condensed state [3]. These TFs act as “master regulators”, promoting the expression of several genes across many signaling pathways, such as differentiation, apoptosis, and cell proliferation. The precise targets of the TFs are controlled by their tissue-specific expression, DNA binding domains, and nucleosome interaction sequences [3]. Additional factors regulating transcriptional activity include: i) cofactors and small molecules that enable TF-DNA recognition and RNA polymerase recruitment [3]; ii) chromatin modifications, such as acetylation, methylation, and phosphorylation, which alter TF access; and iii) methylation of CpG islands in promoter regions that inhibit gene expression [4–5].

There are currently several isolated studies of TF–glycogene interactions, but a systematic “systems-level analysis” is absent. Many of these previous studies are based on discrete glycogene promoter region analysis and reporter assays. These studies have established some notable TF–glycogene relationships, though they are limited to distinct cell types. Examples include the regulation of MGAT5 by ETS2 in NIH3T3 fibroblasts [6], control of the α2-6 sialyltransferases ST6Gal-I/II by hypoxic nuclear factor 1-α (HNF1-α) in HepG2 cells [7], c-JUN-B3GNT8 regulatory relationships in gastric carcinoma cell lines [8], and SP1-B4GALT1 relations in lung cancer A549 cells [9]. A recent study also used computational predictions and wet-lab experiments to determine that ZNF263 is a potential heparin sulfate master regulator [10]. This TF regulates two sulfotransferases, HS3ST1 and HS3ST3A1. The above approaches have limitations: i) they do not consider the cellular epigenetic state that could impact TF binding; ii) proximal regulators are studied, but enhancers present several kilobases away from the transcription state site (TSS) are neglected; and iii) most of these reported TF–glycogene relationships only have partial support in established bioinformatics databases (DBs, see Supporting Information File 1). Thus, these are limited hypothesis-based investigations that do not describe the breadth of the regulatory landscape, based on current knowledge.

**Figure 1:**
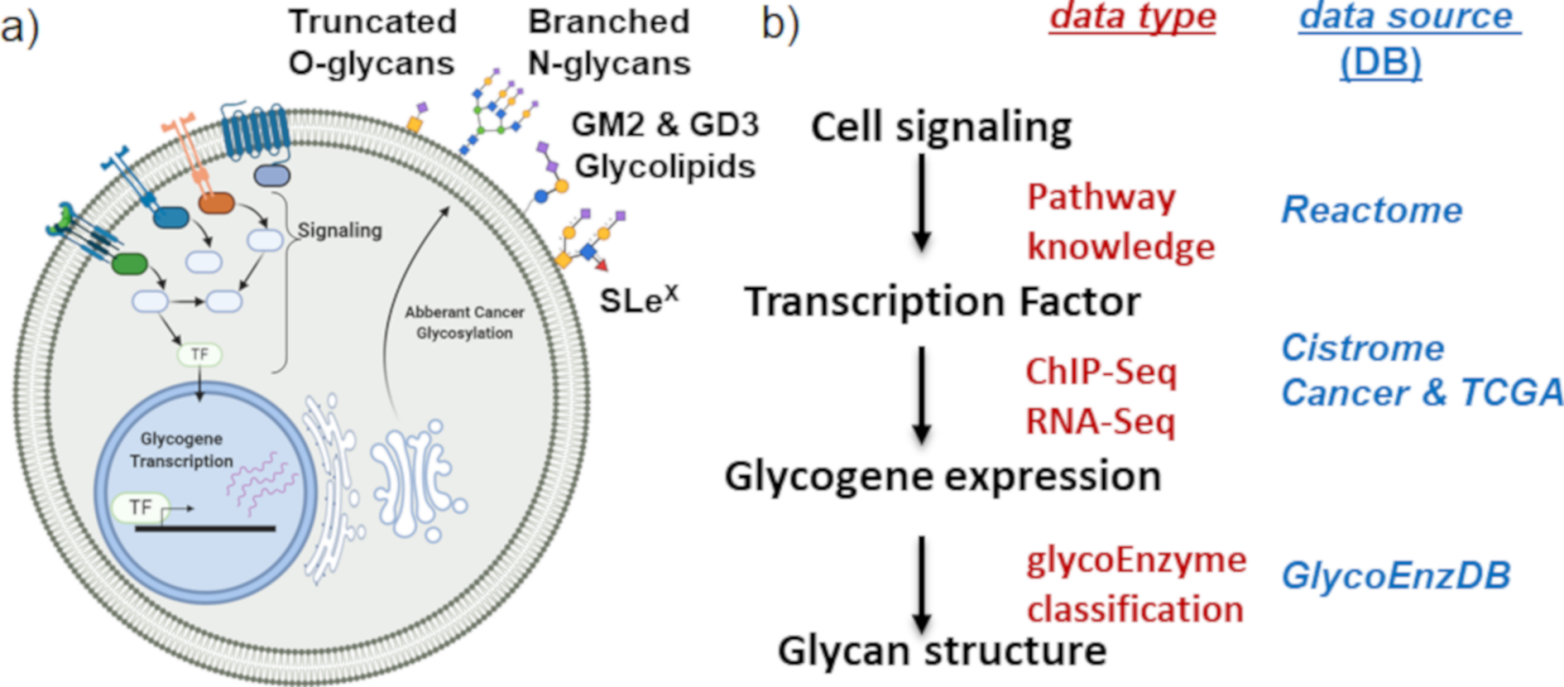
A systems glycobiology framework to link multi-OMICs data. a) Cell signaling proceeds to trigger TF activity. The binding of TFs to sites proximal to the TSS triggers glycogene expression. A complex set of reaction pathways then results in the synthesis of various carbohydrate types, many of which are either secreted or expressed on the cell surface. b) Data available at various resources can establish the link between cell signaling and glycan biosynthesis. The Reactome DB contains cell signaling knowledge. Chip-Seq and RNA-Seq data available at the Cistrome Cancer DB describe the link between the TFs and glycogenes. Pathway curation at GlycoEnzDB establishes the link between glycogenes and glycan structures. Cell illustration created using BioRender (https://biorender.com/).

In the current article, we propose that more global and higher-throughput TF–glycogene relationships under biologically relevant conditions may be discovered using multiomics data mining. To this end, we sought to utilize multiomics experimental datasets and curated pathway DBs to relate cell-specific signaling processes to TFs, TFs to glycogenes, and glycogenes to glycosylation pathways (Figure 1A). These connections were made using data available from Cistrome Cancer DB [11], Reactome DB [12], and by the manual curation of various human glycogenes into pathways at GlycoEnzDB (https://virtualglycome.org/GlycoEnzDB, Figure 1B). Here, the Cistrome Cancer DB uses TF–gene binding data from previously published chromatin immunoprecipitation sequencing (ChIP-Seq) studies for various cell systems and cancer tissue RNA-Seq data from the Cancer Genome Atlas (TCGA) [13]. It provides putative TF–gene relationships for 29 TCGA cancer types provided they satisfy three inclusive criteria: i) TFs should be expressed at a high level in a given tissue; ii) changes in TF gene expression should correlate with RNA changes in target genes; and iii) ChIP-Seq data must support the TF–gene binding proximal to the TSS. Next, knowledge curated in the Reactome DB [12] was used to establish links between TFs and signaling pathways. In the final step, manually curated glycogene classifications were utilized to determine TFs that disproportionately regulate individual glycosylation pathways. It is important to note that the findings from this study represent computational inferences that are yet to be validated in the wet lab. Nevertheless, it provides a systems-based framework for the design and analysis of studies that link TFs to glycosylation pathways and glycan structures.

## Results

### TF–glycogene interaction map and relation to cell signaling pathways

**Figure 2:**
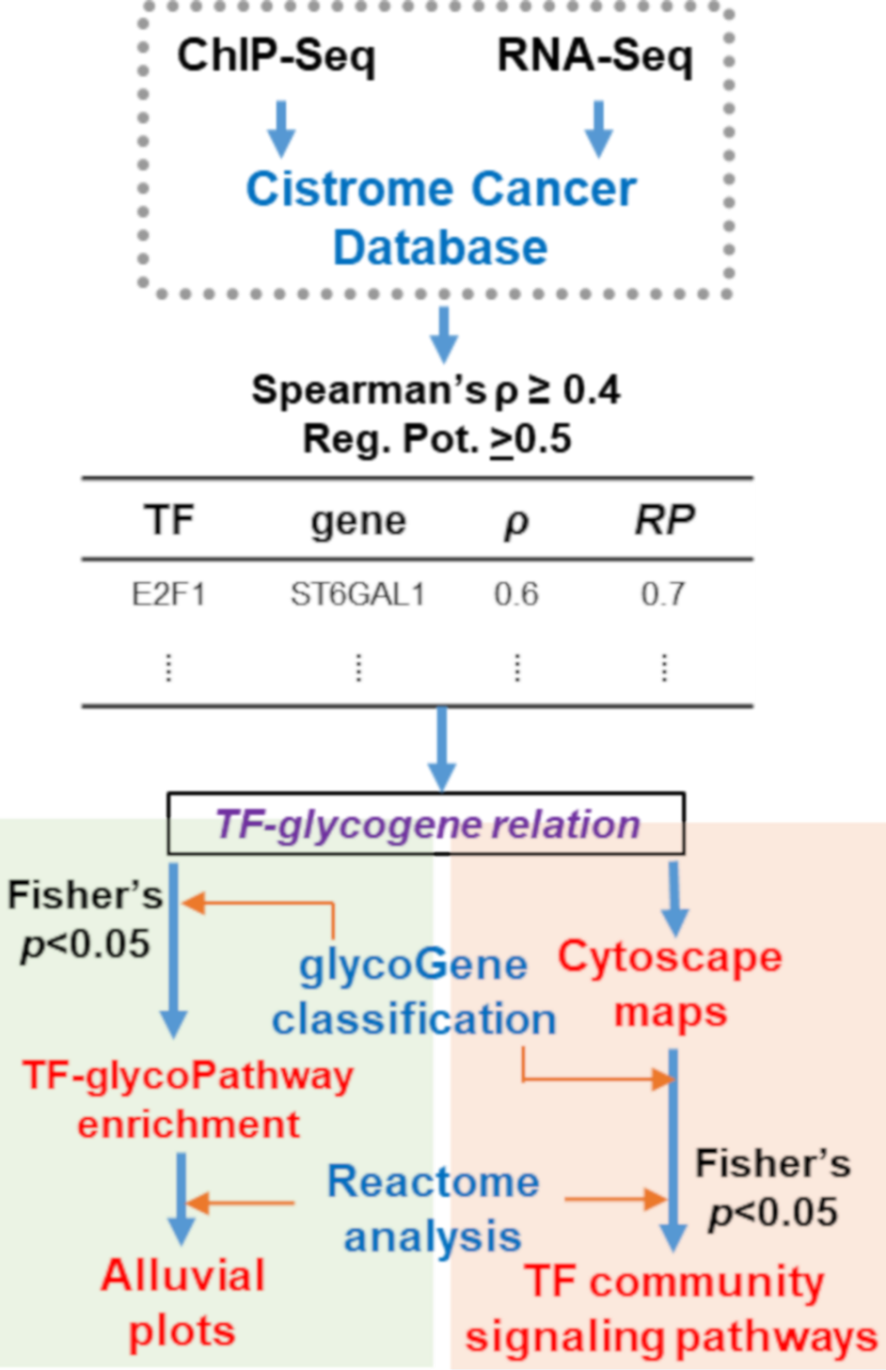
Analysis workflow: ChiP-Seq provides evidence of TF binding to promoter regions with 0 ≤ RP ≤ 1, quantifying the likelihood that this is functionally important. RNA-Seq quantifies Spearman’s correlation (ρ) between TF and gene expression. Filtering these data establishes potential TF–glycogene interactions in specific cancer types. TFs disproportionately regulating specific glycosylation pathways were identified using the above TF–glycogene relationships as well as biochemical knowledge available at GlycoEnzDB (green region). Reactome DB analysis helped to establish cell signaling-TF–glycosylation pathway connectivity that are visualized using alluvial plots. Independently, Cytoscape maps enabled visualization of TF–glycogene relationships in different cancer types (orange region). Clusters in the resulting interactomes were related to pathway maps and signaling processes, and thus developing TF–community signaling pathway relationships.

The article follows a workflow shown in Figure 2. It mines TF–glycosylation pathway relationships from the Cistrome Cancer DB [14], which involves curating TF–gene relationships by integrating ChIP-Seq data from Cistrome DB and RNA-Seq data from TCGA. The Cistrome Cancer DB uses three filtering criteria to determine putative TF–gene relationships: i) The TF should be active in a cancer type, i.e., the reads per kilobase million (RPKM) value in a cancer type must be greater than the median RPKM expression of the TF across all 29 different cancer types; ii) the RNA expression of the TF and target gene should be correlated. To determine this, Cistrome first compares the selected TF–gene correlation with a null distribution computed by randomly selecting 1 million TF–gene pairs. Linear regression and statistical analysis are then performed on the top 5% hits (positive and negative coefficients) to establish TF–gene correlations. This analysis accounts for target gene copy number, tumor purity, and promoter methylation extent; and iii) TF–gene relationships must be supported by ChIP-Seq evidence. Here, a nonlinear weighted sum called regulatory potential (RP) quantifies the strength of TF–gene interactions based on the proximity of TF binding site to the gene TSS and also the number of TF–gene binding interactions based experimentally detected ChIP peaks [15–16].

In the current article, we passed the TF–gene relationships established in Cistrome Cancer DB to identify TFs potentially interacting with 341 glycogenes (Supporting Information File 2, Table S1). The two metrics for this selection were RP ≥ 0.5 and TF–glycogene expression correlation coefficient ρ ≥ 0.4. Such analysis was performed for 29 cancer types listed in Supporting Information File 2 (Table S2). Based on our selected thresholding, the analysis revealed 22,654 potential TF–glycogene interactions. The above data were used for two types of analysis described below. Here, the number of putative TF–glycogene relationships can be tuned by modifying the RP and ρ values.

First, the Fisher’s exact test was used to infer TF–glycogene interactions that may regulate individual glycosylation pathways. This analysis was based on pathway classifications from GlycoEnzDB (Supporting Information File 2, Table S3) that grouped 208 glycogenes into 20 glycosylation pathways/groups. TFs having a disproportionately larger number of relationships with individual glycosylation pathways were determined with respect to all TF–glycogene relationships. Reactome DB was then used to associate these TFs to potential signaling pathways. This resulted in a relationship between cell signaling, TF activity regulation, and glycan structure changes (Supporting Information File 2, Tables S4 and S5). The data are presented as alluvial plots for the 29 cancer types (Supporting Information File 1). Here, the TFs were linked to glycosylation pathways by colored bands if they were found to regulate a disproportionately high fraction of glycogenes belonging to that pathway. Likewise, biological pathways were linked with TFs if that TF was found to be enriched in the biological pathway. Reading these alluvial plots from the left to the right, one can deduce which biological pathways may be potentially involved in regulating TFs, and how these TFs could regulate glycosylation.

Second, we visualized TF–glycogene interactions using Cytoscape maps for each of the cancer types individually (Supporting Information File 3). Regulatory modules were identified with graph clustering methods to identify groups of TFs that regulate common groups of glycogenes. Using our glycosylation pathway definitions, we used Fisher’s exact test to describe what kinds of glycosylation pathways were disproportionately over-represented in each cluster. This analysis revealed 335 glycopathway enrichments in the TF–glycogene communities across the 29 cancer types (Supporting Information File 2, Table S6). Next, we determined, using the Reactome DB overrepresentation API, if the TFs identified in these clusters could be related to specific cell signaling pathways. Here, we noted 901 pathway enrichments across the different cancer types (Supporting Information File 2, Table S7). Common TFs that we observed across all TF–glycogene communities include the TCF and LEF families, FOXO and FOXP, the RUNX family, and IRF family TFs, which were found to regulate diverse glycosylation pathways, such as sialylation pathways, complex N-linked glycan synthesis, as well as chondroitin and dermatan sulfate synthesis.

Overall, the above analysis revealed the existence of communities of TF–glycogene relationships that could be linked to both cell signaling processes and specific glycosylation pathways.

### TF–pathway relationships in breast cancer

We provide a more detailed description of our findings in breast cancer as an example. This disorder appears in 5 unique molecular subtypes based on the PAM50 classification [17]. These include the following: i) normal-like; ii) and iii) luminal A and luminal B, respectively, which overexpress estrogen receptor ESR1; iv) Her2+ tumors, which overexpress the epidermal growth factor receptor (ERBB); and v) basal (triple negative), which express neither ESR1 nor ERBB. Each of these subtypes has unique signaling mechanisms that may contribute to different glycan signatures.

**Figure 3:**
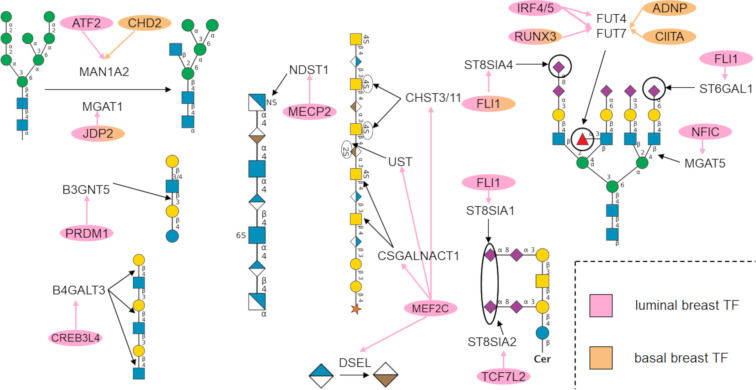
Summary of TFs enriched to glycosylation pathways for luminal and basal breast cancer: The TFs found to be enriched to glycosylation pathways and the glycogenes they regulate are shown in pink for luminal and orange for basal breast cancer. Note that some of the TFs shown above do not appear in the alluvial plots in the subsequent figures because they were not enriched to a signaling pathway in Reactome. The glycans synthesized by the enriched glycogenes are shown in SNFG format [18]. All figures were generated using DrawGlycan-SNFG [19].

In our analysis, TF–glycogene relationships for breast cancer derived by filtering Cistrome Cancer DB were enriched for the glycosylation pathways. Figure 3 summarizes these cancer-related TF–glycosylation pathway relationships for luminal (type A and B together) and basal breast cancer. Here, glycans potentially affected by the enriched TFs are shown in SNFG format [18–19]. The analysis suggests that TF transformations accompanying cancer progression may impact all four major classes of glycans: O- and N-glycans found on glycoproteins, glycosaminoglycans, and glycolipids. Thus, multiple glycan changes may accompany oncological transformation.

### TF–glycogene communities in luminal and basal breast cancer

**Figure 4:**
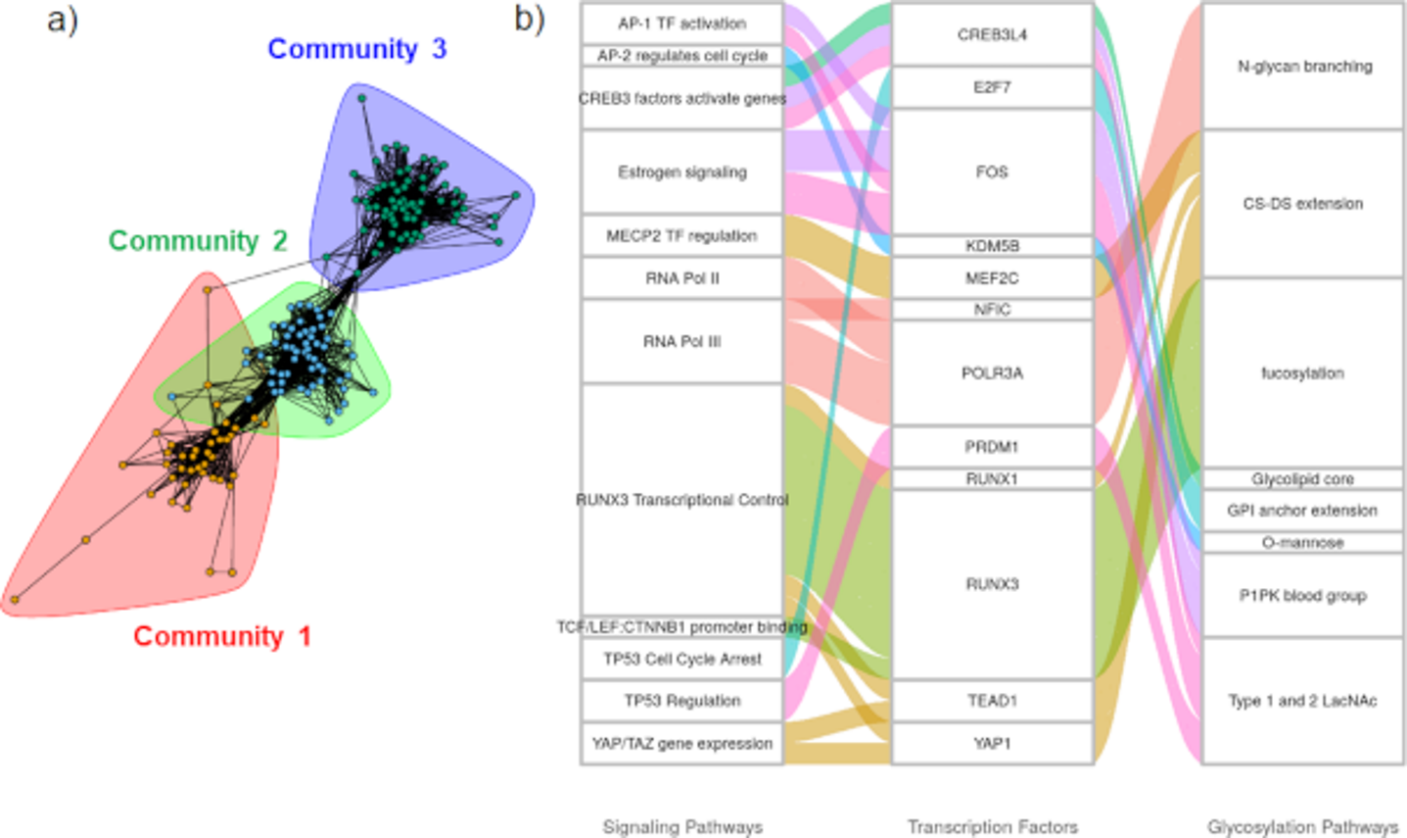
Luminal breast cancer signaling pathway enrichment and glycogene connections. a) TF-to-glycogene communities in luminal breast cancer: Three large TF-to-glycogene communities were discovered in the luminal breast subnetwork. Community 1 was enriched for pathways involving RUNX3, RUNX1, IL-21, and PTEN. Communities 2 and 3 consist primarily of chromatin-modifying enzymes. b) Signaling pathway enrichment analysis for luminal breast cancer: Connections between signaling pathways and TFs found to be statistically significant for luminal breast cancer. Some pathways enriched to TFs were condensed to conserve space. More TF-to-glycogene relationships exist in luminal breast cancer and these can be viewed in the Cytoscape figures (Supporting Information File 1).

Cytoscape plots were generated for luminal breast cancer (Figure 4a). Here, using the bipartite graph community detection methods [20], we identified three large communities of TF–glycogene interactions. The largest community detected in this analysis had TFs enriched for RUNX3 signaling, IL-21 signaling, MECP2, and PTEN regulation. Overrepresentation glycosylation pathway analysis performed on the TFs in this community suggests that these TFs may regulate pathways related to sialylation, hyaluronan synthesis, as well as chondroitin and dermatan sulfate elongation. Here, STAT1, 4, and 5 proteins were enriched in the IL-21 signaling pathway. Luminal breast cancer types are known to express STAT1 and 3 as well as STATs 2 and 4. STAT5 is known to be constitutively active in luminal breast cancer and confers antiapoptotic characteristics to cells [21]. The other two communities detected consisted primarily of chromatin-modifying enzymes. Complex N-linked glycan synthesis and the dolichol pathway were significantly enriched in the second community. In the third community, O-linked mannose and LacdiNAc synthesis were disproportionately regulated. Overall, the pathway maps suggest that chromatin remodeling enzymes could potentially play roles in regulating glycan synthesis in luminal breast cancer.

**Figure 5:**
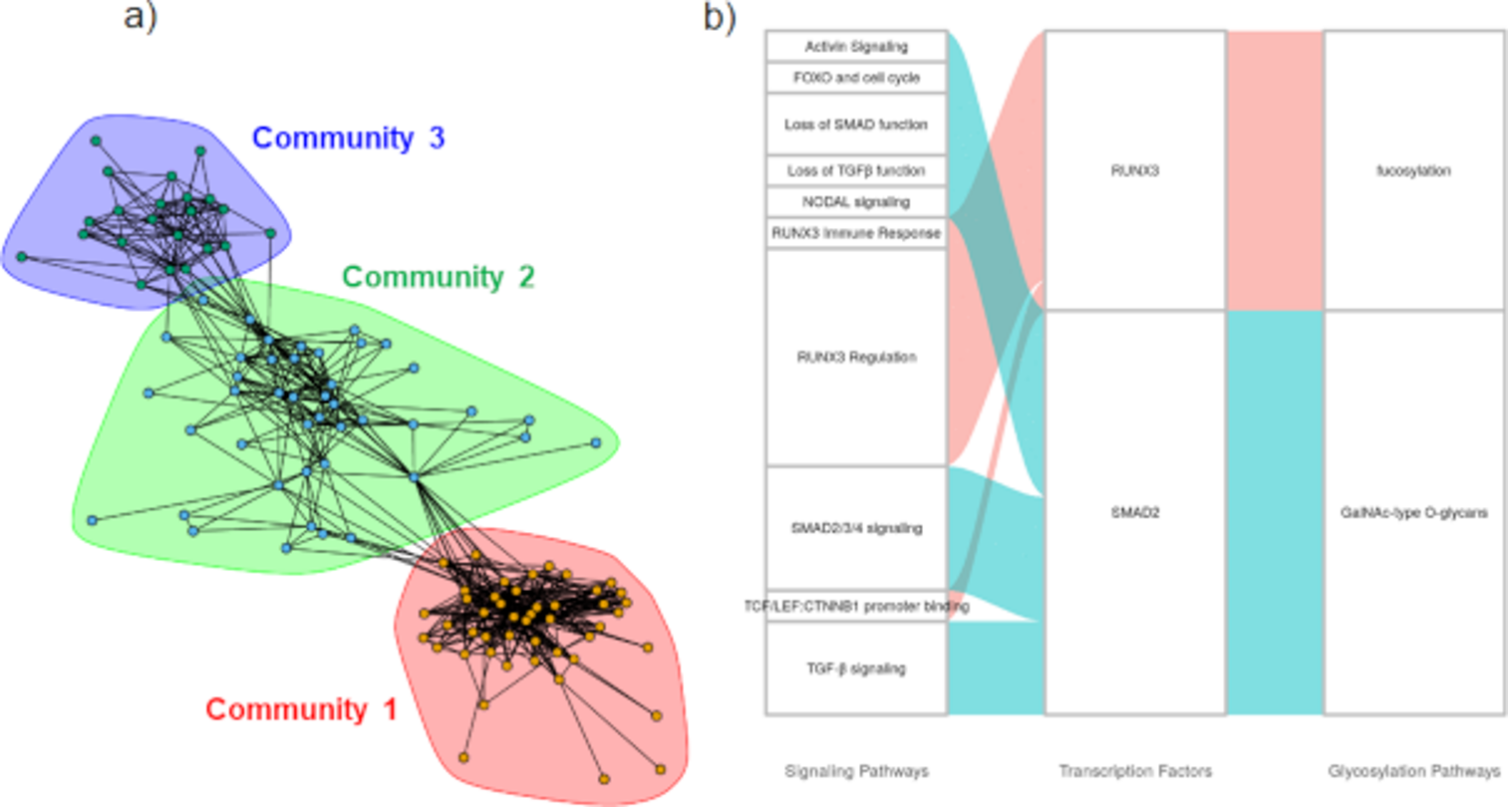
Basal breast cancer signaling pathway enrichments and glycogene connections. a) TF-to-glycogene communities in basal breast cancer: Three large TF-to-glycogene communities were discovered in the basal breast subnetwork. Community 1 has TFs enriched to chromatin-modifying enzymes, and community 2 has TFs enriched to interferon α/β/γ signaling. Community 3 did not have any signaling pathways enriched. b) Signaling pathway enrichment analysis for basal breast cancer: Connections between signaling pathways and TFs found to be statistically significant for basal breast cancer. TFs displayed have been enriched to the displayed glycosylation pathways using Fisher's exact test.

Like luminal, basal breast cancer TF–glycogene relationships were also clustered into three communities. Here, the first community was enriched for chromatin-modifying enzymes, with complex N-linked glycan synthesis being the primary glycosylation pathway being affected (Figure 5a). The second community was enriched for interferon α/β/γ signaling pathways, with interferon regulatory factor (IRF) TFs being enriched. In this regard, the TFs IRF-1 and IRF-5 have been shown to act as tumor suppressors in breast cancer [22–23]. Their loss of function in breast cancer could potentially downregulate O-linked fucosylation. The third community did not exhibit any specific TF pathway enrichments.

### Linking cell signaling to TF and glycogenes for luminal breast cancer

The links between biological signaling pathways, TFs, and glycosylation pathways are shown in alluvial plots for luminal (Figure 4b) and basal breast cancer (Figure 5b), with additional plots provided for additional cancer types in Supporting Information File 1 for luminal breast cancer.

**CREB3L4 and PRDM1 disproportionately affect the type I and II LacNAc pathway in luminal breast cancer:** Our analysis suggests that CREB3L4 (enrichment *p*-value = 0.036) and PRDM1 (enrichment *p*-value = 0.039) may regulate the type 1 and 2 LacNAc pathways. CREB3L4 is known to primarily be expressed in the prostate and some breast cancer cell lines and has been linked to diverse roles involving chromatin organization in spermiogenesis, adipocyte regulation, and dysregulation in prostate cancer [24–25]. It has been found to be upregulated in breast cancer with respect to normal-like. PRDM1, also known as Blimp-1, is a transcriptional repressor, and its upregulation in cancer is known to dysregulate other proteins [26]. The increase poly-LacNAc structures have been shown to play roles in cancer metastasis [27]. CREB3L4 was found to regulate B4GALT3 glycogene (ρ = 0.56, RP = 0.94), which adds galactose in a β1-4 linkage. PRDM1 was found to regulate B3GNT5, which is critical for lacto/neolacto series of glycolipids (ρ = 0.60, RP = 0.84).

**MEF2C disproportionately regulates glycosaminoglycan synthesis pathways:** MEF2C was found to regulate several genes in the chondroitin and dermatan sulfate synthesis pathways (*p* = 0.008). This TF plays roles in development, particularly in the development of neurons and hematopoietic cell differentiation towards myeloid lineages. It is known that MEF2C is directly impacted by TGF-β signaling, and thus increasing the metastatic potential of cancer [28]. MEF2C was found to be inhibited by MECP2 based on Reactome pathway enrichment. Since the glycosaminoglycan elongation pathways positively correlate to MEFC2 expression and MEFC2 is amplified in cancer, it is possible that MECP2 may not be sufficiently expressed to repress MEFC2 in call cancer cells. MEF2C was found to regulate CSGALNACT1 (ρ = 0.66, RP = 0.71), CHST3 (ρ = 0.50, RP = 0.74), CHST11 (ρ = 0.47, RP = 0.84), DSEL (ρ = 0.40, RP = 0.81), and UST (ρ = 0.42, RP = 0.95). Here, CSGALNACT1 is responsible for the addition of GalNAc to glucuronic acid to increase chondroitin polymer length, CHST3, CHST11, and UST are involved in the sulfation of GalNAc and iduronic acid, and DSEL is the epimerase which converts glucuronic acid to iduronic acid in CS/DS chains.

**MECP2 disproportionately regulates heparan sulfate chain elongation:** The MECP2 (enrichment *p*-value = 0.037) was found to positively regulate heparan sulfate elongation. MECP2 regulates gene expression by binding to methylated promoters and then by recruiting chromatin remodeling proteins to condense DNA and repress gene expression [29–30]. MECP2 was found to regulate sulfotransferase NDST1 (ρ = 0.41, RP = 0.67).

### Linking cell signaling to TF and glycogenes for basal breast cancer

Fewer TFs were found to be enriched to signaling pathways in basal breast cancer compared to luminal cancer (Figure 4b). Despite this, there are many other TF–glycosylation pathway enrichments for basal breast cancer available for analysis in Supporting Information File 1. The roles of two enriched TFs and their relation to glycogenes and cancer is elaborated below.

**RUNX3 and fucosylation:** The terminal fucosyltransferase FUT7 (ρ = 0.49, RP = 0.89) was found to be positively regulated by the RUNX3 TF (enrichment *p*-value = 0.033). The RUNX family of TFs (including RUNX1–3), are involved in several developmental processes, including hematopoiesis, immune cell activation, and skeletal development. It was discovered that RUNX3 acts as a tumor suppressor gene in breast cancer. Upon cancer development, the RUNX3 promoter is hypermethylated, leading to reduced TF activity and loss of tumor suppression activity [31]. Our data suggest that this may be associated with a reduction of FUT7 activity, and thus impacting the expression of the sialyl Lewis-X antigens in basal tumors. Sialyl Lewis-X is considered to be an important regulator of cancer metastasis as it binds the selectins on various vascular and blood cell types.

**Regulation of GalNAc-type O-linked glycans by SMAD2:** SMAD2 was found to significantly affect core 1 and 2 O-linked glycan structures (enrichment *p*-value = 0.035). SMAD proteins are activated by TGF-β signaling and bind to DNA to act as cofactors to recruit TFs. SMAD2 has been shown to act as a tumor metastasis suppressor in cell lines [32–33]. This TF was found to regulate GALNT1 (ρ = 0.54, RP = 1.00), which adds GalNAc to serine or threonine residues to being core 1 and 2 O-linked glycan synthesis. Thus, SMAD2 may play a key role in regulating Tn antigen expression in proteins such as MUC-1 that are associated with breast cancer progression.

### Refinement of TF–glycopathway enrichments after false discovery correction

**Figure 6:**
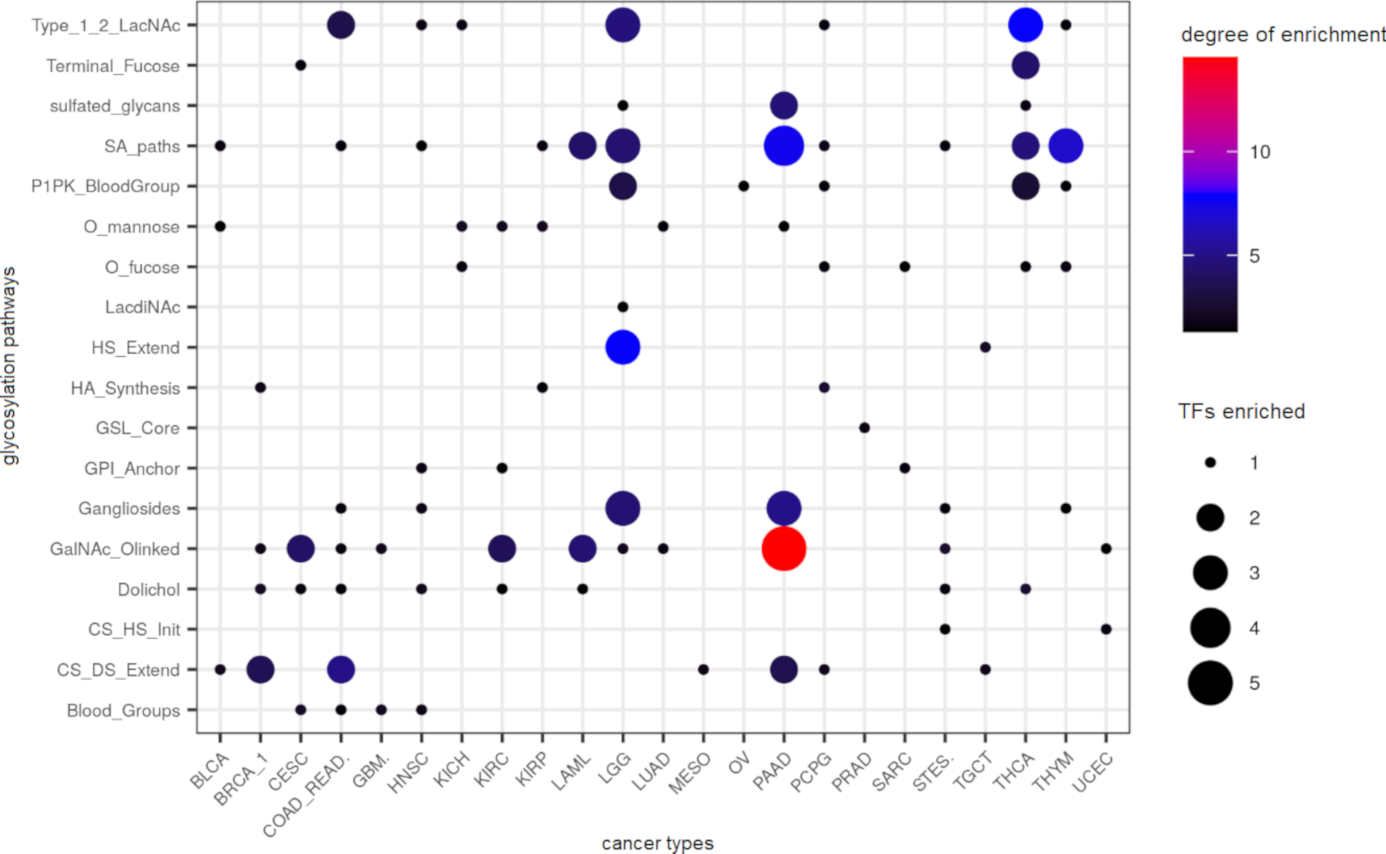
Summary of TF–glycopathway enrichments across all cancer types: TF enrichments to glycopathways across all cancer types are depicted as dots (Fisher’s exact test adjusted *P* < 0.05 for overrepresentation). The dot size corresponds to the number of TFs that were found to regulate the pathway. The degree of regulation is defined as the sum of all −log_10_ (adjusted enrichment *p*-values) across all TFs for a given cancer–glycopathway pair.

The number of enrichments above is high. In order to reduce the findings to a smaller set, we applied the Benjamini–Hochberg correction to our TF–glycopathway enrichments. While possibly reducing false positives, this may also reduce true positives. Nevertheless, after this correction, a total of 121 TF–glycopathway enrichments were found to be statistically significant across all cancer types (Figure 6 and Supporting Information File 2, Table S8). Here, basal breast cancer (BRCA_2), adrenocortical carcinoma (ACC), liver hepatocellular carcinoma (LIHC), lung squamous cell carcinoma (LUSC), and skin cutaneous melanoma (SKCM) did not have any TFs enriched to any glycopathway, and thus are not depicted.

Filtering our TF–glycopathway enrichments illuminates the fact that pancreatic cancer shows a high degree of enrichment to the GalNAc-type O-glycan pathways, which is consistent with our prior experiments [34]. FOXA1 (*P*_adj_ = 0.00096), KLF5 (*P*_adj_ = 0.0012), MECOM (*P*_adj_ = 0.029), and TCF7L2 (*P*_adj_ = 0.000087) were found to regulate several GalNAc transferases. FOXA1 is an important regulatory TF involved in the development of endoderm-derived organs. Upon pancreatic cancer development, FOXA1 expression is known to decrease, which drives the epithelial to mesenchymal transition [35]. Kruppel-like factor 5 (KLF5) is commonly upregulated in several cancer types and promotes pancreatic cancer proliferation by targeting the cell cycle [36]. MECOM (also known as PRDM3) is a nuclear TF known to ablate inflammatory responses and tumorigenesis in pancreatic cancer contexts [37]. Transcription factor 7-like 2 (TCF7L2) is regulated by Wnt β-catenin signaling. This TF is important in gluconeogenesis in the liver, adipogenesis, regulation of hormone synthesis, and pancreas homeostasis. TCF7L2 exhibits polymorphisms which results in loss of function and can promote metastatic phenotypes in colorectal cancer [38]. O-Linked glycosylation via GALNT3 and B3GNT3 has been shown to regulate differentiation of pancreatic cancer stem cells [39]. FOXA1 (RP = 0.97, ρ = 0.49) and KLF5 (RP = 0.71, ρ = 0.68) were found to regulate GALNT3, and KLF5 (RP = 0.98, ρ = 0.67) and TCF7L2 (RP = 0.95, ρ = 0.62) were found to regulate B3GNT3. Since KLF5 and TCF7L2 have been shown to be upregulated in pancreatic cancer stem cells, it would be interesting to validate if GALNT3 and B3GNT3 are driven by any of these TFs.

## Discussion

In the current analysis, we mined public high-throughput ChIP-Seq and RNA-Seq data to identify putative TF–glycogene relationships across 29 different cancer types. Approximately three glycogenes were regulated by a given TF based on our filtering criteria, with this number ranging from 1–10. These findings are tissue-specific, as TF and glycogene expression vary widely among the different cell types. The analysis also suggests putative TF–glycogene interactions that disproportionately impact specific glycosylation pathways. Knowing which TF regulates which glycogene and pathway in a context-dependent manner can provide insight as to how signaling pathways contribute to altered glycan structures in diseases such as diabetes and cancer. Thus, this work represents a rich starting point for wet-lab validation and glycoinformatics DB construction.

Visualizing TF–glycogene interaction networks revealed communities of glycogenes in each cancer type. The presence of chromatin-modifying enzymes in large regulatory communities in both luminal and basal breast cancer suggests a role of epigenetics in glycogene regulation. To date, a systems-level investigation evaluating the epigenetic states of cell systems on the resulting glycome has not been performed. Our results suggest that complex N-linked branching and glycosylation may be sensitive to these processes. The signaling pathways enriched in the largest community in luminal breast cancer were reflected in our pathway enrichment findings. RUNX3, interleukin signaling, and the involvement of MECP2 regulation were all found to disproportionately regulate sialic acid and GAG synthesis pathways.

Several of the TFs enriched to glycosylation pathways were either regulated by or involved in TGF-β signaling and Wnt β-catenin signaling. These TFs primarily affected glycosaminoglycan synthesis pathways, sialylation, and type-2 LacNAc synthesis. Cell cycle and metabolic regulatory TFs were shown to regulate some glycogenes involved in the dolichol pathway. The crosstalk between cell cycle and glycosylation is not well explored and may potentially be important for understanding N-linked glycosylation flux in cancer. Some TFs were found to interact with methyl CpG-binding TFs when regulating glycosaminoglycan proteins, implicating methylation as a possible modulator of glycosylation in cancer.

Our TF–glycogene relationships, mined from Cistrome Cancer DB, represent a starting point for experimentally discovering the TFs regulating glycosylation. The findings would likely vary between cell types, and thus additional efforts are necessary before a wet-lab-validated framework emerges. Orthogonal datasets containing other ChIP-Seq and omics data may also enhance in silico validation. Some examples include: i) data from the Gene Transcription Regulatory Database (GTRD) [40], which has analyzed publicly available ChIP-Seq data with multiple algorithms to systematically catalog TF–gene relationships across several organisms and cellular contexts; ii) the Regulatory Circuits DB [41], which relies on the activity of promoter and enhancer regions through cap analysis of gene expression (CAGE), TF motif instances, and expression quantitative trait loci (eQTL) to evaluate weights (evidence scores) for TF–gene isoform relationships; and iii) integration of TF-binding motifs, protein–protein interactions, and coexpression networks using data from GTEx and a method called PANDAS [42]. Such analyses represent next steps in this project, as extensive data harmonization is required for cross-platform validation. Care should be taken when integrating these data, however, as the kind of omics data, degree of experimental evidence, and the statistical approaches taken by other investigators can influence the set of TF–gene relationships found. In addition to in silico validation, perturbational experiments, such as performing CRISRP-Cas9 knockouts with single-cell RNA-Seq, followed by glycomics/glycoproteomics-based mass spectrometry, would further support the proposed TF–glycogene relationships [43].

Some caveats in our analysis are important to note. First, we only used selected values of RP and ρ to filter TF–glycogene relationships from the Cistrome Cancer DB. Further studies are needed in order to determine how the selected thresholds affect the discovered relationships. A full list of TF–glycogene relationships found Cistrome Cancer DB are provided in Supporting Information File 2 (Table S9) for readers to test alternative thresholds. Second, the glycogenes in individual pathways in this article were classified using current knowledge of glycobiology. Different classification methods meant to address different glycosylation pathways may result in different TF–glycopathway enrichments [44]. Third, while Cistrome Cancer DB systematically filters TF–gene relationships based on ChIP-Seq and RNA-Seq evidence, the DB has some biases. In one aspect, only TFs that were considered to be sufficiently expressed were considered in this analysis. Lower expressed TFs that may also be functional are excluded. Additionally, while RNA-Seq relationships in Cistrome Cancer DB are selected based on the specific tissue type, supporting ChIP-Seq evidence is not cell-type-specific. Regardless of these limitations, the current study presents a framework for thinking in the glycosciences, so that knowledge of genes and transcripts can be linked to glycans and their function [2].

## Conclusion

A majority of current studies in the Glycoscience field use experimental data and curations related to glycans only. Fewer investigations examine the links between the glycans, glycogenes and glycosylation pathways, and other nonglyco datasets. We set out to identify these relationships by mining publicly-available data. Using this, we describe putative regulatory relationships between TFs and glycogenes across 29 cancer types. Some TFs appear to regulate glycogenes in communities, indicating potential cross-talk across pathways in regulating glycosylation. The communities varied with cancer type, even in a single tissue, suggesting that these TF–glycogene interactions are dynamic in nature. Groups of TFs enriched to glycosylation pathways were also associated with signaling pathways. Thus, a connection between cell signaling, TF activity and glycosylation begins to emerge. Overall, the putative TF–glycosylation pathway enrichments found here represent the starting point for wet-lab and orthogonal dataset validation. Such studies could enhance our fundamental understanding of glycosylation pathway regulation, and lead to novel ways to control the glycogenes and glycan structures during health and disease.

## Experimental

### Glycogene-pathway classification

A list of 208 unique glycogenes involved in 20 different glycosylation pathways were used in this work (Supporting Information File 2, Table S3). These data were collated from GlycoEnzDB (https://virtualglycome.org/GlycoEnzDB), with original data coming from various sources in literature [45–46]. The following is a summary of the pathways studied and the enzymes involved:

**1) Glycolipid core:** The enzymes in this group are involved in the biosynthesis of the glucosylceramide (GlcCer) and galactosylceramide (GalCer) lipid core. Here, the GlcCer core is formed by the UDP-glucose:ceramide glucosyltransferase (UGCG), which transfers the first glucose. Following this, lactosylceramide is formed by the action of the β1-4GalT activity of B4GalT5 (and possibly also B4GalT3, 4, and 6). The GalCer core is typically structurally small and is made by UDP-Gal:ceramide galactosyltransferase (UGT8). These structures can be further sulfated by GAL3ST1 or sialylated by ST3GAL5.

**2) P1-Pk blood group:** The Pk, P1, and P antigens are synthesized on lactosylceramide glycolipid core. The activity of α1-4GalT (A4GALT) on this core results in the Pk antigen, followed by β1-3GalNAcT (B3GALNT1) to form the P antigen. The P1 antigen, on the other hand, is formed by the sequential action of β1-3GlcNAcT (B3GNT5), β1-4GalT (B4GALT1-6), and α1-4GalT (A4GALT) on the glycolipid core.

**3) Gangliosides:** This pathway encompasses all glycogenes responsible for synthesizing a/b/c gangliosides. UGCG is included to consider the addition of glucose to ceramide. ST3GAL5 and ST8SIA enzymes are added to take the core ganglioside structures to the a, b, and c levels. B4GALTs and B4GALNT1 are included to account for ganglioside elongation. Decoration of the gangliosides with sialic acid occurs using ST6GALNAC3-6 and also ST8SIA1/3/5.

**4) Dolichol pathway:** This results in the formation of the dolichol-linked 14-monosaccharide precursor oligosaccharide. This glycan is cotranslationally transferred en bloc onto Asn-X-Ser/Thr sites of the newly synthesized protein as it enters the endoplasmic reticulum. The enzymes involved is such synthesis include the ALG (asparagine-linked N-glycosylation) enzymes and additional proteins (part of OSTA and OSTB) involved in the transfer of the glycan to the nascent protein.

**5) Complex N-glycans:** This pathway includes glycogenes responsible for processing the N-linked precursor structure emerging from the dolichol pathway into complex structures. Enzymes involved include mannosidases, glucosidases, some enzymes facilitating protein folding, and also enzymes that direct acid hydrolases to the lysosome.

**6) N-glycan branching:** These glycogenes are responsible for the addition of GlcNAc to processed N-linked glycan structures. These include all the MGAT enzymes.

**7) GalNAc-type O-glycans:** O-linked glycans are attached to serine (Ser) or threonine (Thr) on peptides, where GalNAc is the root carbohydrate. This is mediated by a family of about 20 Golgi-resident polypeptide *N*-acetylgalactosaminyltransferases (ppGalNAcTs or GALNTs). Core 1 structures result from the attachment of β1-3 linked galactose to the core GalNAc using C1GALT1 and the corresponding chaperone C1GALT1C1. Core 2 structures then form upon addition of β1-6-linked GlcNAc by GCNT1. Modifications of core 3 and core 4 glycans can occur during disease, and thus this classification includes core 3-forming B3GNT6 and core 4-forming GCNT3. Other O-glycan core types are rare in nature.

**8) Chondroitin sulfate and heparan sulfate initiation:** Chondroitin and heparan sulfate glycosaminoglycans all have a common core carbohydrate sequence attaching them to the corresponding proteins. These are constructed by the activity of specific xylotransferases (XYLT1 and XYLT2), galactosyltransferses B4GALT7 and B3GALT6 that sequentially add two galactose residues to xylose, and the glucuronyltransferase B3GAT3 that adds glucuronic acid to the terminal galactose. Also involved in the formation of this core is FAM20B, a kinase that 2-*O*-phosphorylates xylose. At this point, the addition of GalNAc to GlcA by CSGALNACT1 and 2 results in the initiation of chondroitin sulfate chains. The attachment of GlcNAc by EXTL3 to the same GlcA results in heparan sulfates.

**9) Chondroitin sulfate and dermatan sulfate extension:** Chondroitin sulfates and dermatan sulfates are extended via the addition of GalNAc-GlcA repeat units. This is catalyzed by CSGALNACT1, which is better suited for the initial GalNAc attachment, followed by CSGALNACT2, which is preferred for synthesizing disaccharide repeats. CHSY1, CHSY3, CHPF, and CHPF2 all exhibit dual β1-3GlcAT and β1-4GlcAT activity. Additional enzymes mediate sulfation. Epimerization of glucuronic acid to iduronic acid by DSE and DSEL results in the conversion of chondroitin sulfates to dermatan sulfates.

**10) Heparan sulfate extension:** EXT1 and EXT2 both have GlcUA and GlcNAc transferase activities and are together responsible for HS chain polymerization. EXTL1–3 are additional enzymes with GlcNAc transferase activity that facilitate heparin sulfate biosynthesis. Additional enzymes that are critical for heparin sulfate function include the HS2/3/6ST sulfotransferases, the GlcA epimerase GLCE, and additional enzymes mediating N-sulfation (i.e., NDSTs).

**11) Hyaluronan synthesis:** This pathway consists of the three hyaluronan synthases, HAS1–3.

**12) Glycophosphatidylinositol (GPI) anchor extension:** This pathway includes glycogenes responsible for the synthesis of GPI-anchored proteins in the ER. This involves the synthesis of a glycan–lipid precursor that is en bloc transferred to proteins.

**13) O-Mannose:** This is initiated by the addition of mannose to Ser/Thr using POMT1 or POMT2. β1-2 or β1-4 GlcNAc linkages can then be made using POMGNT1 or POMGNT2 to yield M1 or M3 O-linked mannose structures, respectively. MGAT5B can facilitate β1-4 GlcNAc linkage onto the M1 structure to yield the M2 core. Additional carbohydrates typically found on complex N-linked glycan antennae can then be attached. In particular, such extensions may be initiated by members of the B4GALT family or B3GALNT2. Specific variants are noted on α-dystroglycans.

**14) O-linked fucose:** This pathway includes POFUT1, the enzyme responsible for the addition of fucose to Ser/Thr residues. MFNG, LFNG, and RFNG can attach β3GlcNAc to this fucose.

**15) Type 1 and 2 LacNAc:** These enzymes help construct either Galβ1-3GlcNAc (type 1) or Galβ1-4GlcNAc (type 2) lactosamine chains on antennae of N-linked glycan, O-linked glycans, and glycolipids. Also included are GCNT1–3 that can facilitate formation of I-branches on N-glycans.

**16) Sialylation:** This group encompasses all kinds of sialyltransferases: ST6GAL, ST3GAL, ST8SIA, and ST6GALNACs. Enrichments to this pathway capture overall increase in sialylation regardless of context.

**17) Fucosylation:** these include α1-2 (FUT1, 2) and α1-3 (FUT3–7, 9) fucosyltransferases that can act on N-glycans, O-glycans and glycolipids.

**18) ABO blood group synthesis:** these are enzymes involved in the biosynthesis of ABO antigens.

**19) LacDiNAc:** glycogenes involved in the synthesis of LacDiNac structures.

**20) Sulfated glycan epitopes:** this includes the enzymes attaching sulfate to different types of carbohydrates.

### Mining TF–glycogene relationships in Cistrome Cancer DB

Regulatory potential and gene correlation data were downloaded from the Cistrome Cancer DB in tab-delimited form (http://cistrome.org/CistromeCancer/CancerTarget/) [14]. TF–gene relationships were filtered for the 341 glycogenes in this article (Supporting Information File 2, Table S1). In total, the full dataset contained 45,238 TF-to-glycogene relationships, including relational data for 570 unique TFs found in the 29 cancer systems across all the glycogenes. Positive regulatory relationships between TFs and glycogenes were selected based on RP ≥ 0.5 and ρ ≥ 0.4 (Figure 2). This filtering resulted in 22,654 TF–glycogene relationships including 526 unique TFs across 29 cancer types.

Cytoscape was used to visualize TF–glycogene regulatory relationships [47]. To achieve this, all TF–glycogene relationship data were loaded into Cytoscape as a network. These data were filtered based on RP and ρ thresholds defined previously. A binding potential (BP) score was computed by taking the product of RP and ρ for each TF–glycogene relationship. TF–glycogene relationships for each cancer type were separated into subnetworks. The Prefuse Force Directed Layout algorithm in Cytoscape was used to arrange nodes in each cancer subnetwork. The closeness of nodes to one another is weighted by 1-BP. Thus, nodes with high BPs will be placed closer together, whereas smaller BPs will be placed further away. Since there are two classes of nodes (TFs and glycogenes), we treated TF–glycogene networks as bipartite and applied the corresponding procedure for community detection [20]. Firstly, the bipartite TF–glycogene graphs are projected into two different unipartite graphs, where TFs and glycogenes are placed into separate graphs. The edge weights connecting TFs is computed as the number of shared glycogenes they regulate. The TF unipartite graph was then subjected to a greedy modularity optimization-based approach implemented in the igraph R package [48]. TF–glycogene interactions in each community were subjected to overrepresentation analyses to identify enriched signaling and glycosylation pathways.

### Relating TF–glycogene interactions to glycosylation and signaling pathways

A one-sided Fisher’s exact test was applied to determine if a particular TF disproportionately regulates one of the 20 glycosylation pathways described in Supporting Information File 2 (Table S3). Input data to the test consisted of all TF–gene interactions that passed the RP and ρ thresholds for the cancer type being analyzed. TFs were considered to be disproportionately regulating a glycosylation pathway if Fisher’s exact test resulted in a *p*-value ≤ 0.05. These *p*-values were then adjusted using the Benjamini–Hochberg method to identify the strongest enrichments across all cancer types.

TFs enriched to glycosylation pathways were associated with putative regulatory pathways using the Reactome DB overrepresentation analysis API, which also uses Fisher’s exact test, to associate the TFs with signaling pathways [12]. Signaling pathway enrichments with adjusted *p*(FDR) < 0.1 were kept. A high *p*-value cutoff was chosen to allow users to gain a high-level perspective as to what potential pathways may be regulating enriched TFs. The connection between cell signaling pathways and TFs and that between the TFs and glycosylation pathways were visualized using alluvial plots generated using the R package ggalluvial. Only signaling pathways with <30 members are presented for brevity. A comprehensive listing of enriched signaling pathways is available in Supporting Information File 2 (Table S5).

## Supporting Information

File 1 Comparison of wet-lab studies and entries in DBs as well as Alluvial plots for all cancer types.

File 2 Cistrome Cancer TF-to-glycogene subnetworks.

File 3 Supplementary tables.
