# Supplementary material for: A systems-based framework to computationally describe putative transcription factors and signaling pathways regulating glycan biosynthesis
Source: Beilstein J Org Chem. 2021 Jul 22;17:1712–24. doi: 10.3762/bjoc.17.119 (PMC8313979; doi:10.3762/bjoc.17.119)
Supplement: File 1 — Comparison of wet-lab studies and entries in DBs as well as Alluvial plots for all cancer types. [file Beilstein_J_Org_Chem-17-1712-s001.pdf]

BEILSTEIN JOURNAL OF ORGANIC CHEMISTRY

Supporting Information

for

A systems-based framework to computationally describe putative transcription factors and signaling pathways regulating glycan biosynthesis

Theodore Groth, Rudiyanto Gunawan and Sriram Neelamegham

Beilstein J. Org. Chem. **2021,** *17,* 1712–1724. doi:10.3762/bjoc.17.119

Comparison of wet-lab studies and entries in DBs as well as Alluvial plots for all cancer types

License and Terms: This is a supporting information file under the terms of the Creative Commons Attribution License (https://creativecommons.org/licenses/by/4.0). Please note that the reuse, redistribution and reproduction in particular requires that the author(s) and source are credited and that individual graphics may be subject to special legal provisions.
The license is subject to the *Beilstein Journal of Organic Chemistry* terms and conditions: (https://www.beilstein-journals.org/bjoc/terms)

Level of agreement between wet-lab studies and corresponding entries in the databases.

Proposition: We determined if findings reported in literature (see Introduction section) have corresponding entries in bioinformatics databases that present TF–(glyco)gene relationships. Such comparisons were made with Cistrome DB, GTRD, and Regulatory Circuits DB (see table below). The footnote explains the metrics used for this comparison.

Conclusion: The analysis concludes that experimental data reported in literature only have partial support in currently established databases. The lack of agreement was due to several reasons: i) Sometimes, the TF was not included in the curation of the database; ii) sometimes, the TF in question was filtered out due to the database criteria for establishing TF–gene relationships; and iii) the putative TF–glycogene relationship is present in some cell systems, but not the cell system reported in literature. This suggests that the same TF–glycogene relationship may not hold for all cell types.

Table S1: To what degree are interactions reported in literature observed in various databases?

literature reports[a]			GTRD[b]	Cistrome database[c]	Regulatory Circuits[d]
glycogene	TF	expt. cell system	cell system where TF–glycogene relationship was found in database		
B4GALT1	SP1	A549	A375 (malignant melanoma); GM12878 (female B-cells lymphoblastoid cell line); HCT-116 (colon carcinoma); HEK293 (embryonic kidney); HUES64 (embryonic stem cells); liver	SP1 not present in DB	107 tissue-specific relationships with conf $\geq$0.1, significant
ST6Gal1	HNF1a	HepG2	not found (N.F.)	HNF1A not present in DB	2 with conf$\geq$0.1, (hepatocyte, spinal cord)
MGAT5	ETS2	NIH3T3	N.F.	N.F.	N.F.
ST6Gal2	HNF1a	HepG2	N.F.	HNF1A not in DB	N.F.
B3GNT8	c-JUN	Gastric Carcinoma	A549 (lung carcinoma); Dexamethasone-treated HUVEC-C. Metaclusters are absent in gastric carcinoma	N.F.	N.F.
HS3ST1	ZNF263	HeLa	HEK293 (embryonic kidney) K562 (myelogenous leukemia). Metaclusters are absent in HeLa.	ZNF263 not in DB	ZNF263 not in DB
HS3ST3A1	ZNF263	HeLa	K562 (myelogenous leukemia). Metaclusters are absent in HeLa	ZNF263 not in DB	ZNF263 not in DB

[a]Reported in literature (see Introduction section in the main manuscript). [b]GTRD: The GTRD curates TF binding sites on the genome by employing 4 different ChIP-Seq peak calling algorithms. Any binding region identified by more than one ChIP-Seq peak finding algorithm is designated as a metacluster. Thus, metaclusters represent TF binding regions with support from multiple ChIP-Seq peak calling algorithms. In our analysis, we parsed glycogene transcriptional start sites (TSSs) from Ensembl BioMart. Only transcripts with an Ensembl support level of 1 were used. To identify regulating TFs, the distance between the center of a metacluster and a glycogene TSS was computed. TF metaclusters identified 2 kb upstream from any glycogene TSS constituted a TF–glycogene relationship. This yielded a total 5,190 TF–glycogene interactions across 302 biological systems. [c]Cistrome DB: TF–glycogene relationships were parsed from Cistrome Cancer DB by picking regulatory potential ≥ 0.5 and correlation values ≥ 0.4. This yielded 22,654 TF–glycogene interactions in Cistrome Cancer DB. These thresholds were selected to find long- and short-range TF–glycogene interactions. [d]Regulatory Circuits: TF–glycogene relationships with a confidence score ≥ 0.1 were parsed from each of the 394 tissue-specific networks from Regulatory Circuits. This yielded 66,071 relationships across all 394 tissue systems.

Details: The following is a detailed explanation of our findings for each interaction and cell system:

SP1→B4GALT1 in A549 cell line:
i) Cistrome DB: This relationship was not found in Cistrome DB since this TF was not included in the database. It is unclear if SP1 has been filtered from the database due to low tumor expression specificity, or if it was not included in the analysis.
ii) GTRD: SP1 was found in the promoter region of 6 different cell systems: A375, GM12878, HCT-116, HEK293, HUES64, and the “liver” system. In the A549 data (wet-lab data are reported in literature for this system), however, the SP1→B4GALT1 relation was not supported by the multiple ChIP-Seq algorithm used by GTRD. In this regard, while the SP1→B4GALT1 relation was reported in A549 using the MACS algorithm in one study. Three other algorithms used in GTRD (GEM, PICS, SISSRS) do not support a SP1→B4GALT1 relationship in A549. Thus, the SP1→B4GALT1 metacluster for A549 is not reported in the GTRD.
iii) Regulatory Circuits: This relation was observed in large cell lung carcinoma cell line network, which is derived from NCI-H460 and IA-LM cell lines (confidence threshold = 0.105). Since this network does not contain the A549 relationship, we conclude that SP1→B4GALT1 relation does not exist in A549 in Regulatory Circuits.

HNF1A→ST6Gal1/2 in HepG2 cell line:
i) Cistrome DB: HNF1A was not found in the downloaded Cistrome DB dataset. It was also not found upon searching the online database portal (http://cistrome.org/CistromeCancer/CancerTarget/). This may be due to low expression of this TF across TCGA cancers.
ii) GTRD: HNF1A data were available for 6 different cell systems across 9 distinct experiments. None of these systems supported a HNF1A→ST6GAL1 or HNF1A→ST6GAL1 metacluster relationship. Peaks called by GEM, MACS2, PICS, and SISSRS were loaded into the UCSC genome browser to see if individual algorithms supported the HNF1→ST6GAL1/2 relationship. No peak calling algorithm found HNF1A binding in the ST6GAL1 promoter.
iii) Regulatory Circuits: The HNF1A→ST6GAL1 relationship exists in the “hepatocyte” (confidence = 0.110) and “spinal cord adult” (confidence = 0.103) networks. However, the HNF1A→ST6GAL1 confidence threshold for three HepG2 CAGE sequencing replicates, called “hepatocellular carcinoma cell line”, was low and below our cutoff criterion (confidence value = 0.013).

ETS2→MGAT5 in NIH3T3 cell line:
i) Cistrome DB: ETS2 was not found in the downloaded Cistrome DB, nor was it searchable from the database portal (http://cistrome.org/CistromeCancer/CancerTarget/). This could be due to the lack of expression in the TCGA dataset.
ii) GTRD: No metaclusters were identified supporting the ETS2→MGAT5 relationship.

iii) Regulatory Circuits: No ETS2→MGAT5 relationships were found in any of the tissue-specific networks in Regulatory Circuits
c-JUN—B3GNT8 in gastric carcinoma:
i) Cistrome DB: None of the correlation data or regulatory potential data present in Cistrome DB lists B3GNT8 being regulated by c-JUN. It is unclear for what reason this relationship was omitted.
ii) GTRD: dexamethasone-treated HUVEC-C cells and A549 cells support the c-JUN→B3GNT8 relationship. But this relationship is missing in gastric carcinoma systems, tissues and cell lines, and thus there is no evidence supporting this relationship.
iii) Regulatory Circuits: The B3GNT8 gene is absent in the regulatory networks of this database (see the other supplementary data files). Thus, we cannot assess validity of this relationship in Regulatory Circuits.

HS3ST1/HS3ST3A1→ZNF263 in HeLa cells:
i) Cistrome DB: ZNF263 was not found in the downloaded Cistrome DB, nor was it searchable from their database portal (http://cistrome.org/CistromeCancer/CancerTarget/). Low expression of this TF across TCGA cancers could potentially explain its absence.
ii) GTRD: ZNF263 has metaclusters in the promoter region of HS3ST1 in both HEK293, as well as K562 cells. ZFN263 also has metaclusters in the promoter region of HS3ST3A1 in K562. However, this relationship was absent in HeLa cells.
iii) Regulatory Circuits: ZNF263 was not considered in the construction of tissue-specific networks. There is no position weight matrix (PWM) information present for ZNF263 (see Regulatory Circuits supplemental material).

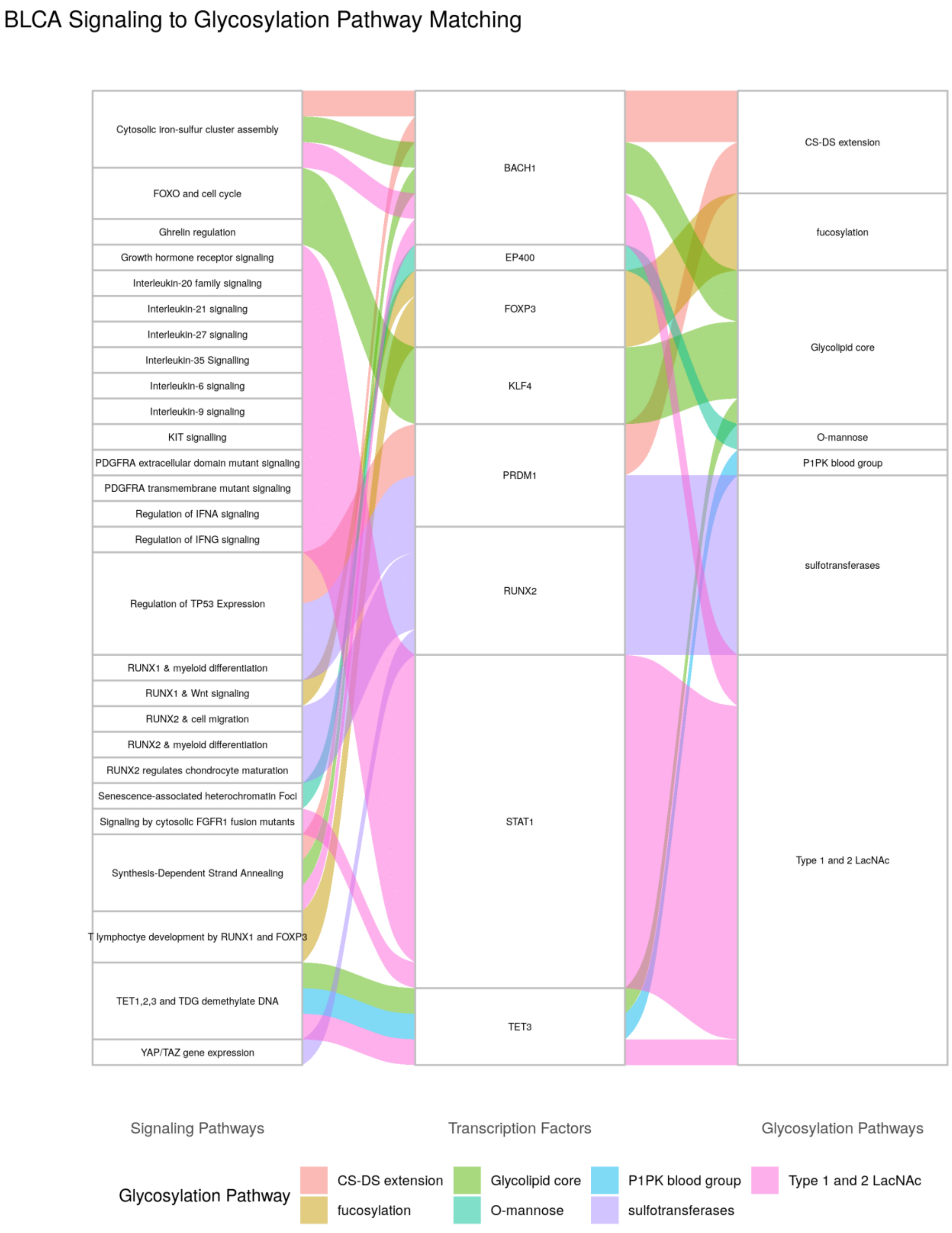
BLCA Signaling to Glycosylation Pathway Matching
Cytosolic iron-sulfur cluster assembly
FOXO and cell cycle
Ghrelin regulation
Growth hormone receptor signaling
Interleukin-20 family signaling
Interleukin-21 signaling
Interleukin-27 signaling
Interleukin-35 Signalling
Interleukin-6 signaling
Interleukin-9 signaling
KIT signalling
PDGFRA extracellular domain mutant signaling
PDGFRA transmembrane mutant signaling
Regulation of IFNA signaling
Regulation of IFNG signaling
Regulation of TP53 Expression
RUNX1 & myeloid differentiation
RUNX1 & Wnt signaling
RUNX2 & cell migration
RUNX2 & myeloid differentiation
RUNX2 regulates chondrocyte maturation
Senescence-associated heterochromatin Foci
Signaling by cytosolic FGFR1 fusion mutants
Synthesis-Dependent Strand Annealing
T lymphoctye development by RUNX1 and FOXP3
TET1,2,3 and TDG demethylate DNA
YAP/TAZ gene expression
BACH1
EP400
FOXP3
KLF4
PRDM1
RUNX2
STAT1
TET3
CS-DS extension
fucosylation
Glycolipid core
O-mannose
P1PK blood group
sulfotransferases
Type 1 and 2 LacNAc
Signaling Pathways
Transcription Factors
Glycosylation Pathways
Glycosylation Pathway
CS-DS extension
fucosylation
Glycolipid core
O-mannose
P1PK blood group
sulfotransferases
Type 1 and 2 LacNAc

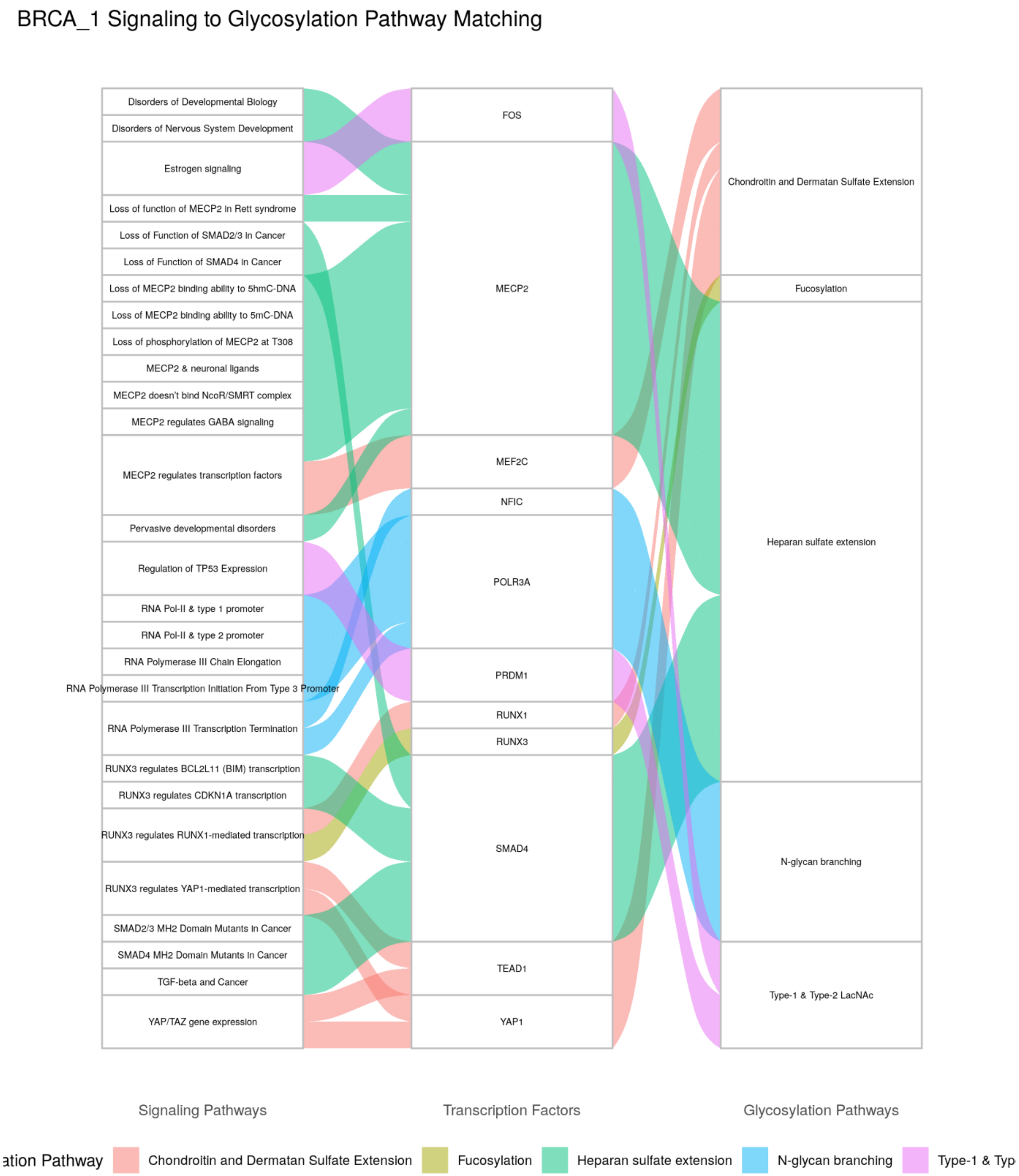
BRCA_1 Signaling to Glycosylation Pathway Matching
Disorders of Developmental Biology
Disorders of Nervous System Development
Estrogen signaling
Loss of function of MECP2 in Rett syndrome
Loss of Function of SMAD2/3 in Cancer
Loss of Function of SMAD4 in Cancer
Loss of MECP2 binding ability to 5hmC-DNA
Loss of MECP2 binding ability to 5mC-DNA
Loss of phosphorylation of MECP2 at T308
MECP2 & neuronal ligands
MECP2 doesn't bind NcoR/SMRT complex
MECP2 regulates GABA signaling
MECP2 regulates transcription factors
Pervasive developmental disorders
Regulation of TP53 Expression
RNA Pol-II & type 1 promoter
RNA Pol-II & type 2 promoter
RNA Polymerase III Chain Elongation
RNA Polymerase III Transcription Initiation From Type 3 Promoter
RNA Polymerase III Transcription Termination
RUNX3 regulates BCL2L11 (BIM) transcription
RUNX3 regulates CDKN1A transcription
RUNX3 regulates RUNX1-mediated transcription
RUNX3 regulates YAP1-mediated transcription
SMAD2/3 MH2 Domain Mutants in Cancer
SMAD4 MH2 Domain Mutants in Cancer
TGF-beta and Cancer
YAP/TAZ gene expression
FOS
MECP2
MEF2C
NFIC
POLR3A
PRDM1
RUNX1
RUNX3
SMAD4
TEAD1
YAP1
Chondroitin and Dermatan Sulfate Extension
Fucosylation
Heparan sulfate extension
N-glycan branching
Type-1 & Type-2 LacNAc
Signaling Pathways
Transcription Factors
Glycosylation Pathways
ation Pathway
Chondroitin and Dermatan Sulfate Extension
Fucosylation
Heparan sulfate extension
N-glycan branching
Type-1 & Typ

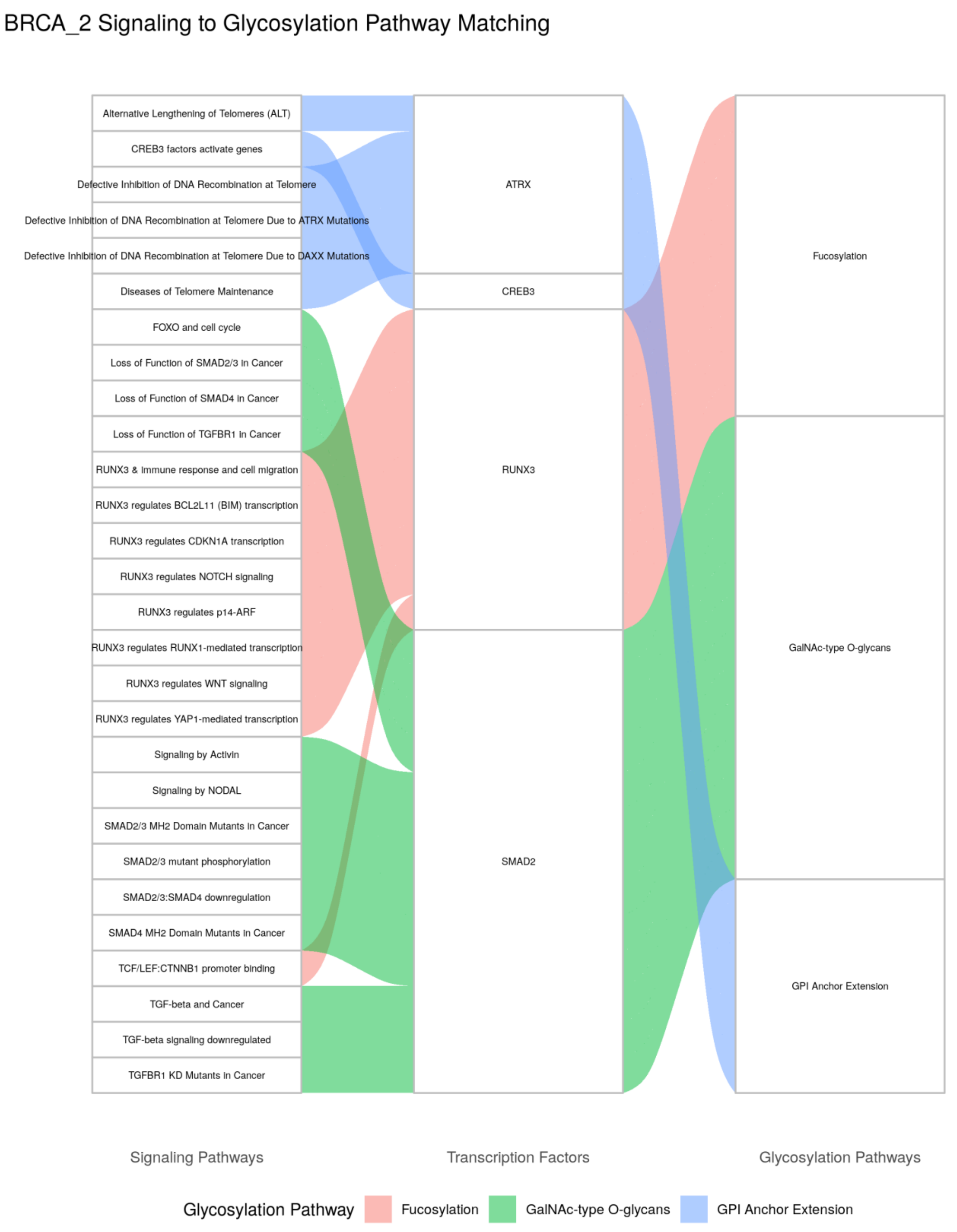
BRCA_2 Signaling to Glycosylation Pathway Matching
Alternative Lengthening of Telomeres (ALT)
CREB3 factors activate genes
Defective Inhibition of DNA Recombination at Telomere
Defective Inhibition of DNA Recombination at Telomere Due to ATRX Mutations
Defective Inhibition of DNA Recombination at Telomere Due to DAXX Mutations
Diseases of Telomere Maintenance
FOXO and cell cycle
Loss of Function of SMAD2/3 in Cancer
Loss of Function of SMAD4 in Cancer
Loss of Function of TGFBR1 in Cancer
RUNX3 & immune response and cell migration
RUNX3 regulates BCL2L11 (BIM) transcription
RUNX3 regulates CDKN1A transcription
RUNX3 regulates NOTCH signaling
RUNX3 regulates p14-ARF
RUNX3 regulates RUNX1-mediated transcription
RUNX3 regulates WNT signaling
RUNX3 regulates YAP1-mediated transcription
Signaling by Activin
Signaling by NODAL
SMAD2/3 MH2 Domain Mutants in Cancer
SMAD2/3 mutant phosphorylation
SMAD2/3:SMAD4 downregulation
SMAD4 MH2 Domain Mutants in Cancer
TCF/LEF:CTNNB1 promoter binding
TGF-beta and Cancer
TGF-beta signaling downregulated
TGFBR1 KD Mutants in Cancer
ATRX
CREB3
RUNX3
SMAD2
Fucosylation
GalNAc-type O-glycans
GPI Anchor Extension
Signaling Pathways
Transcription Factors
Glycosylation Pathways
Glycosylation Pathway
Fucosylation
GalNAc-type O-glycans
GPI Anchor Extension

CESC Signaling to Glycosylation Pathway Matching

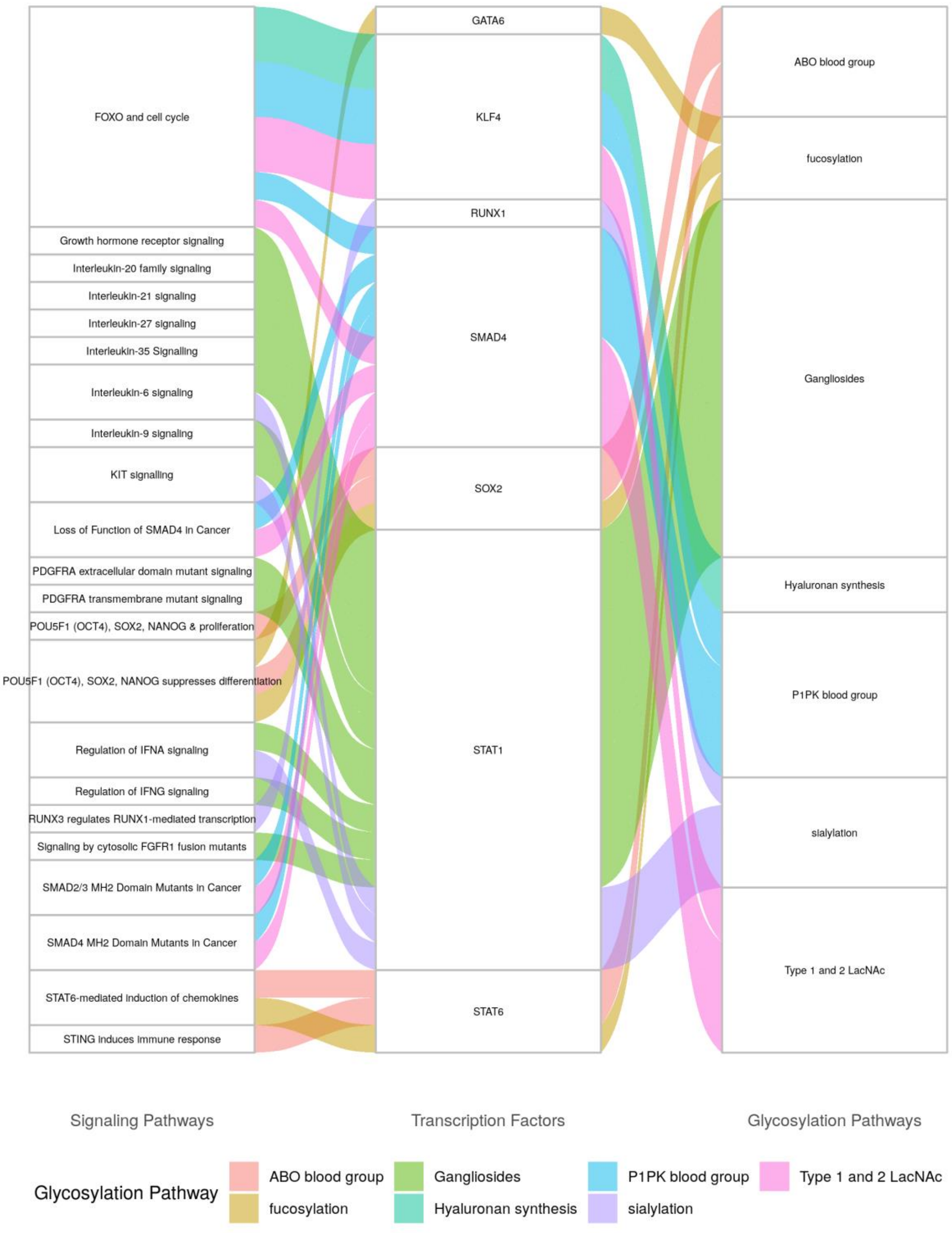

COAD_READ. Signaling to Glycosylation Pathway Matching

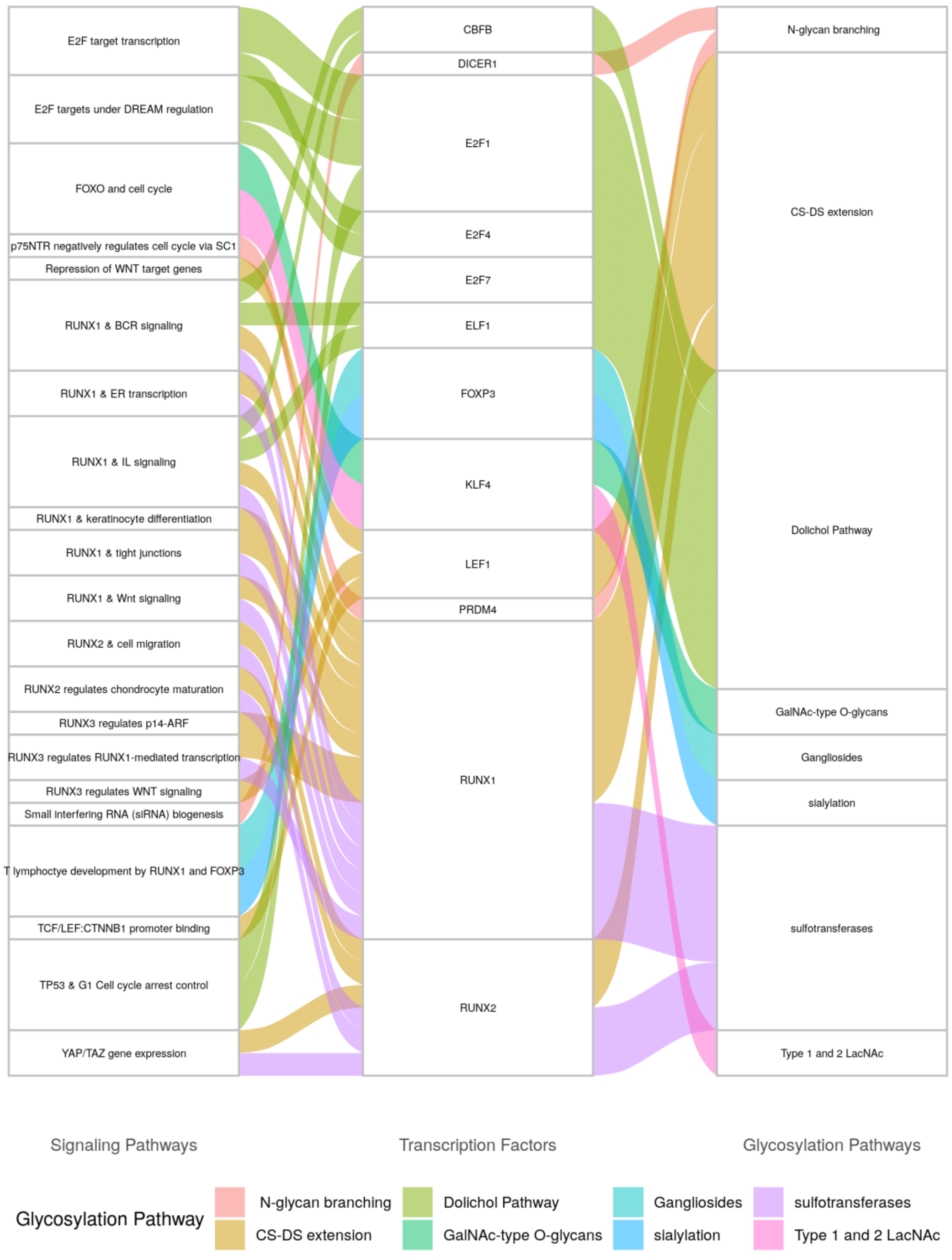

GBM. Signaling to Glycosylation Pathway Matching

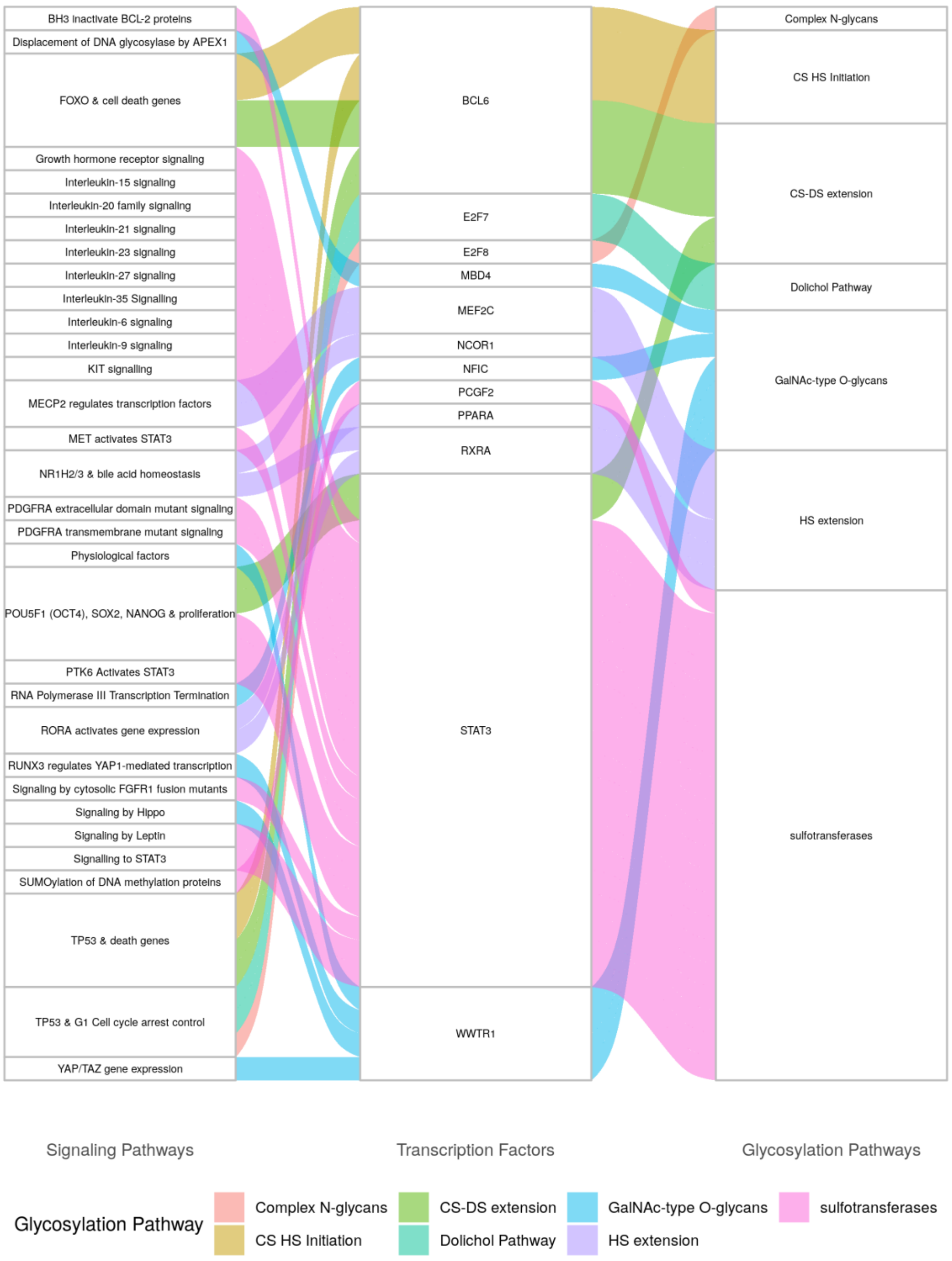

HNSC Signaling to Glycosylation Pathway Matching

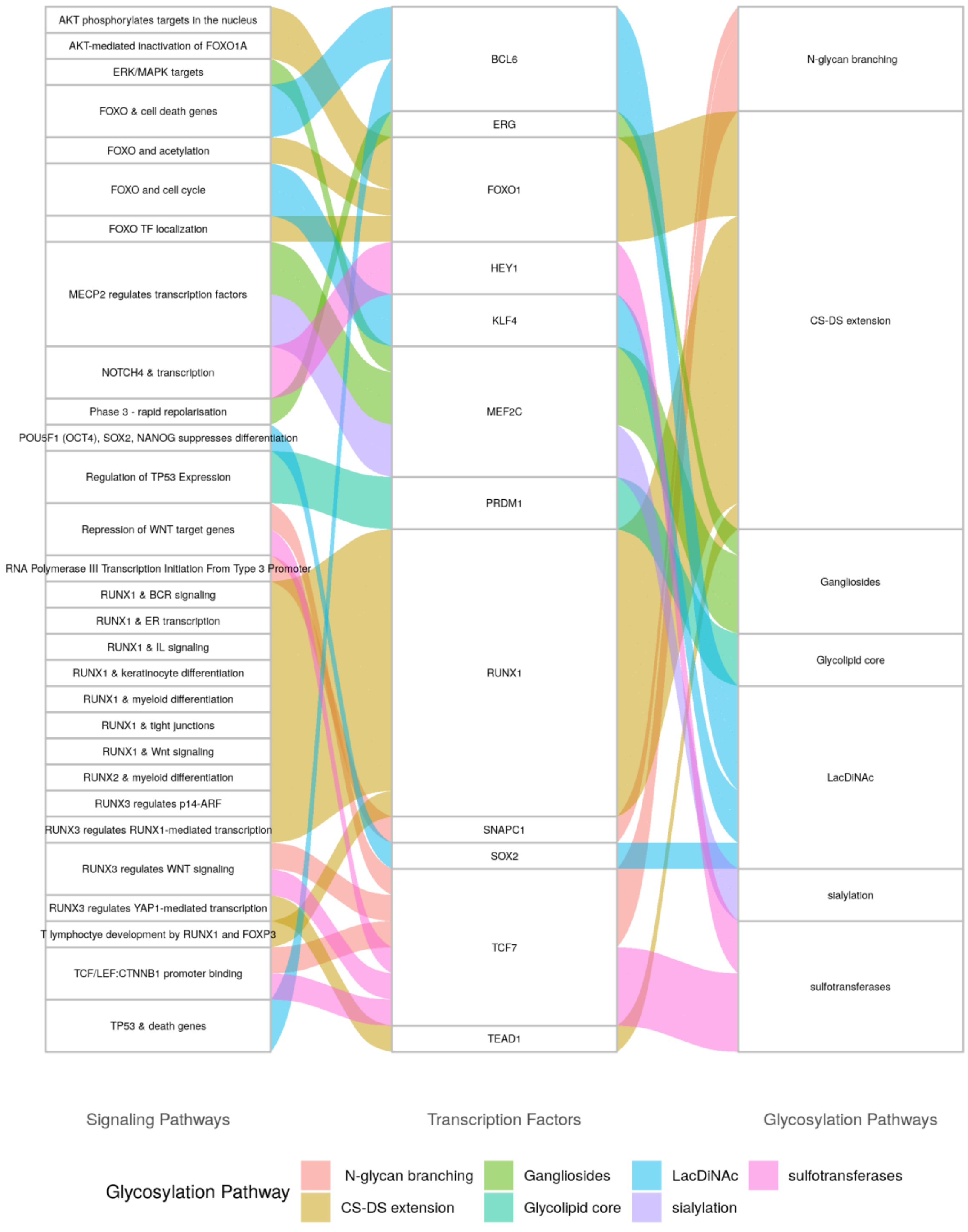

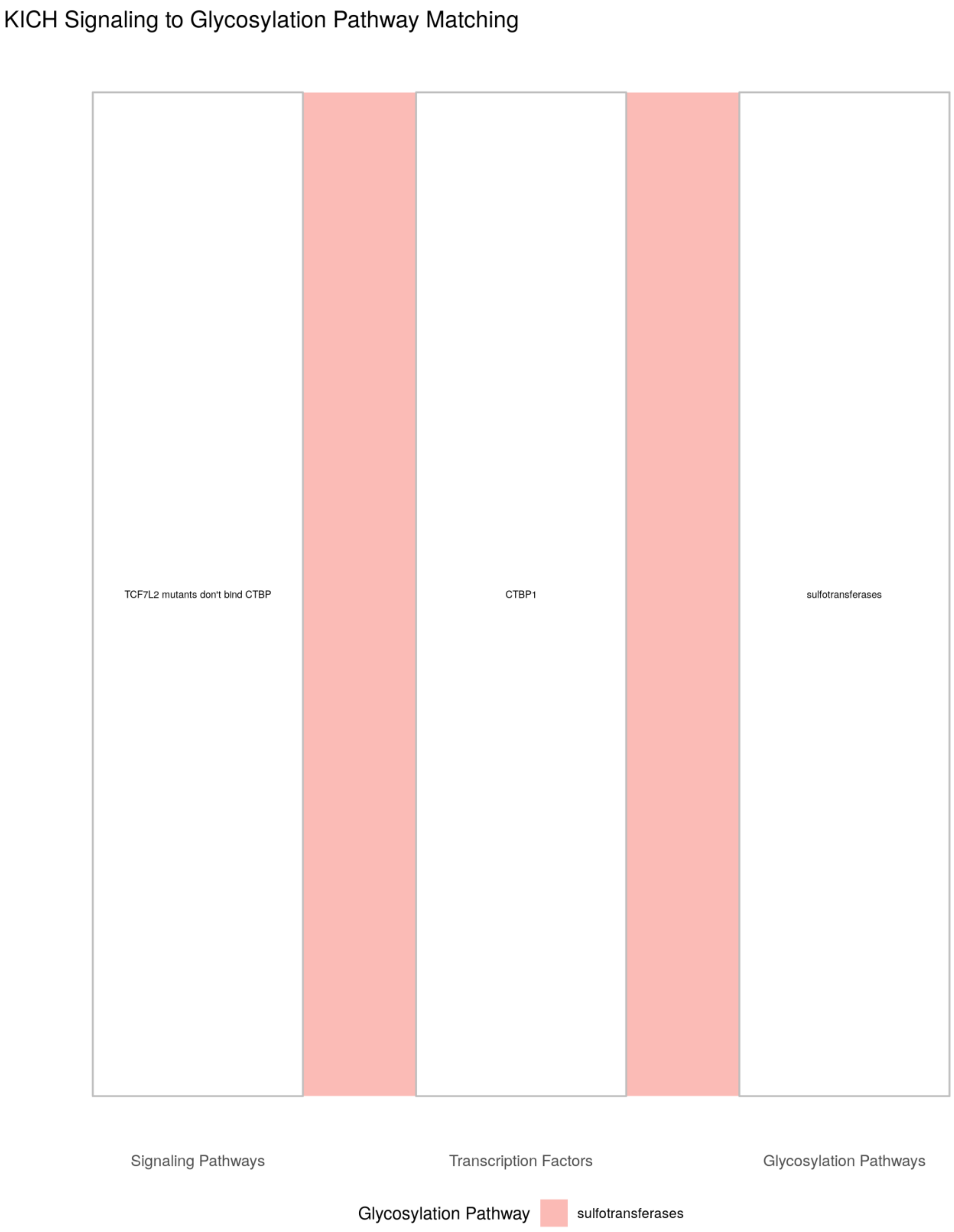
KICH Signaling to Glycosylation Pathway Matching
TCF7L2 mutants don't bind CTBP
CTBP1
sulfotransferases
Signaling Pathways
Transcription Factors
Glycosylation Pathways
Glycosylation Pathway
sulfotransferases

KIRC Signaling to Glycosylation Pathway Matching

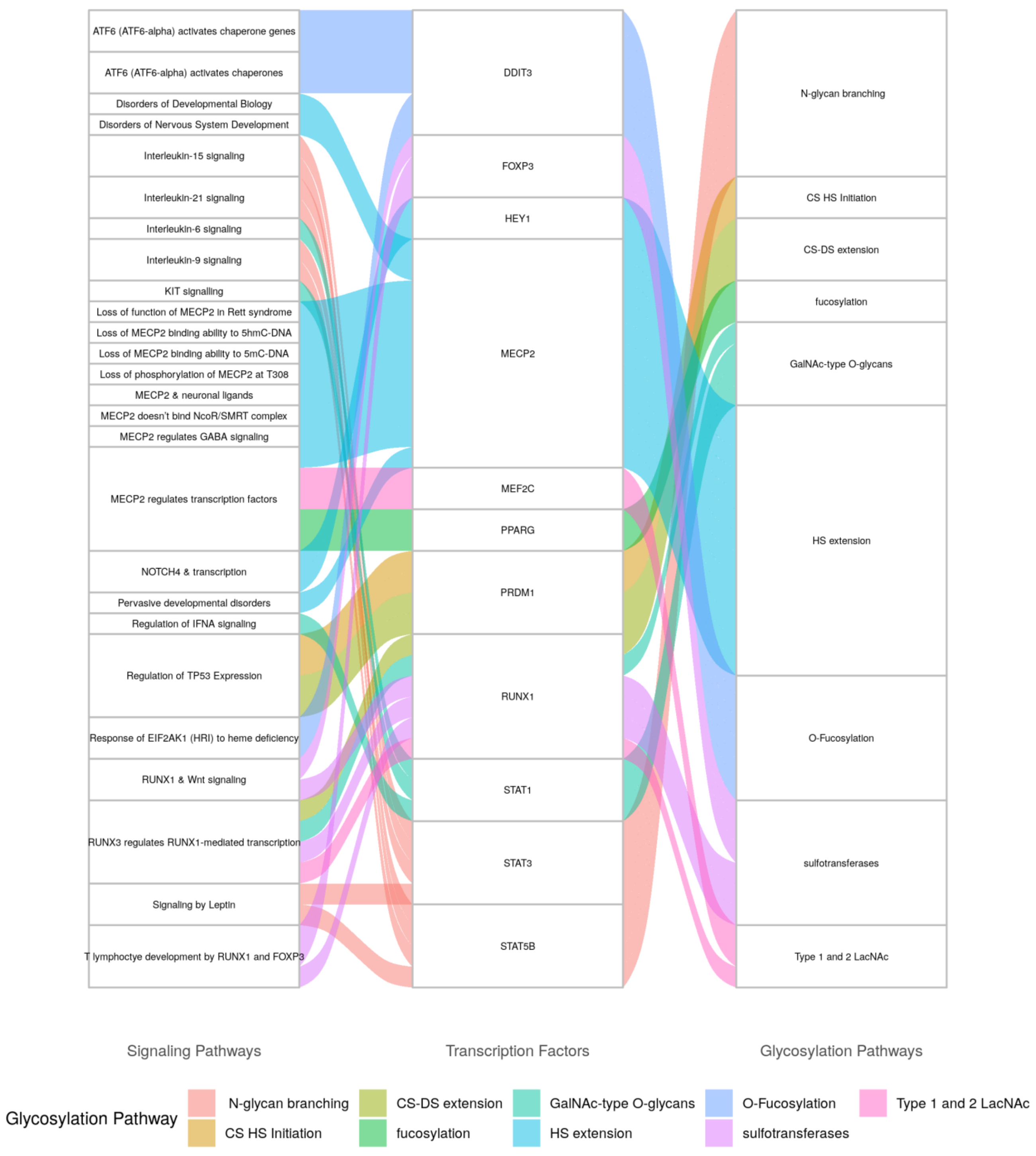

KIRP Signaling to Glycosylation Pathway Matching

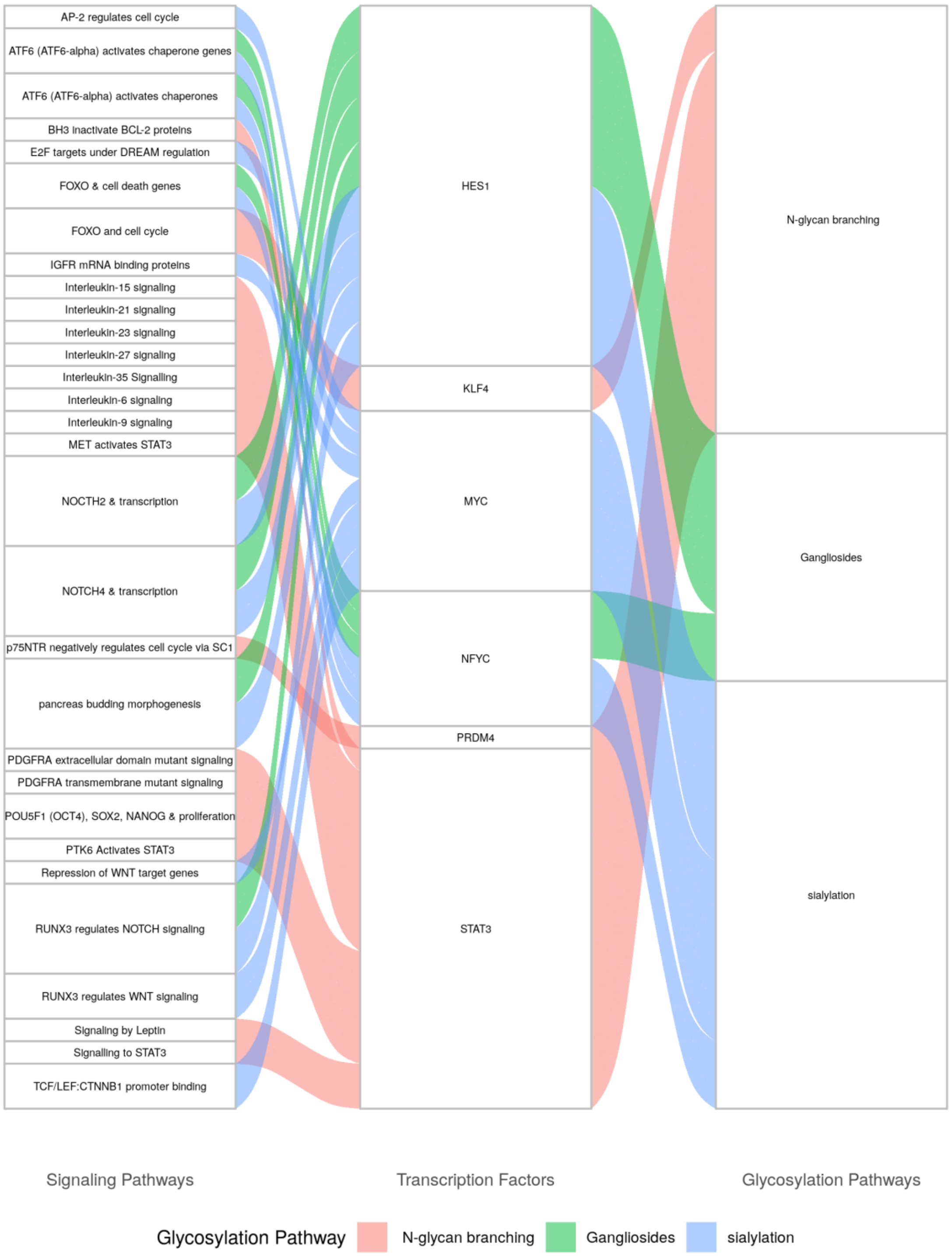

LAML Signaling to Glycosylation Pathway Matching

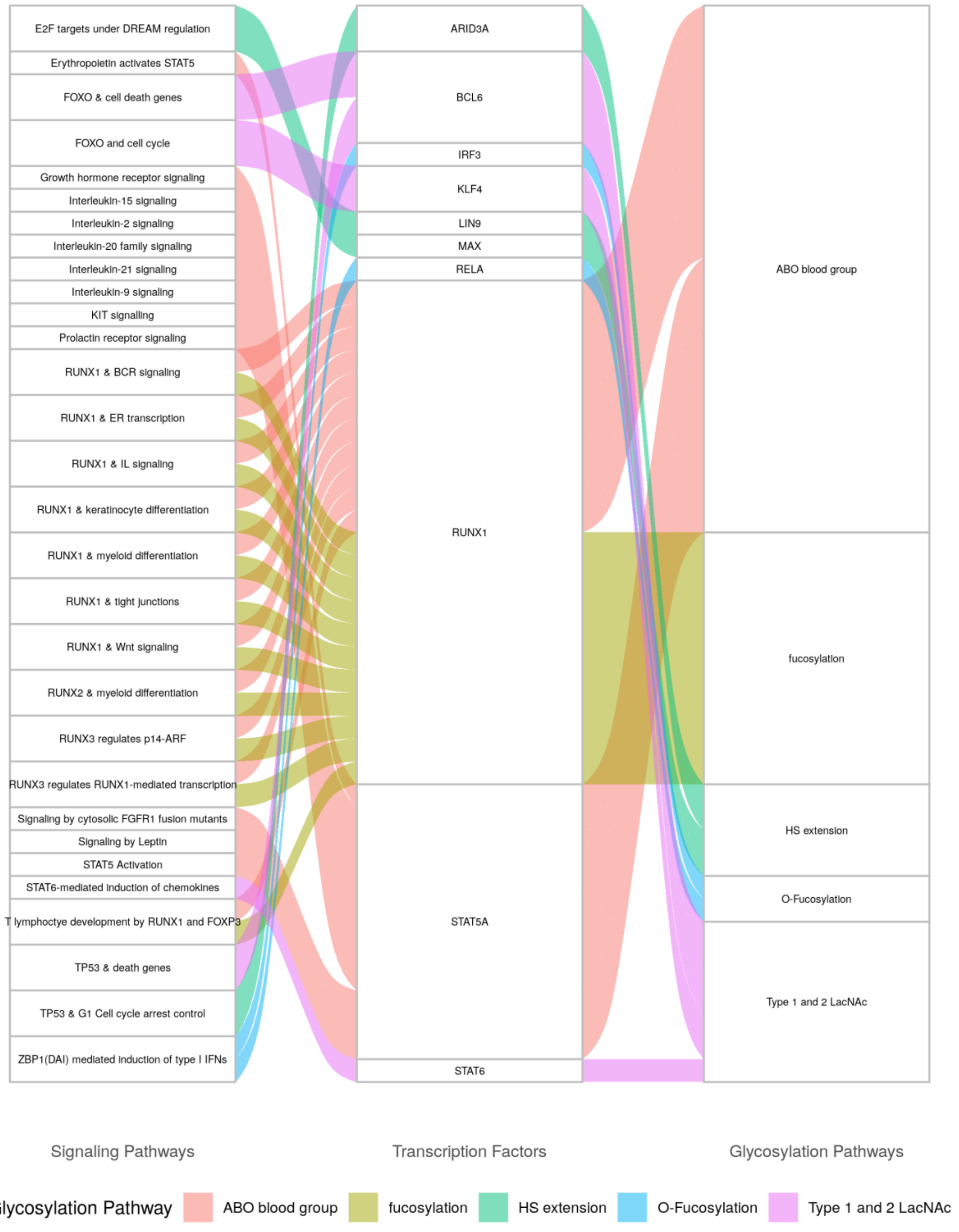

LGG Signaling to Glycosylation Pathway Matching

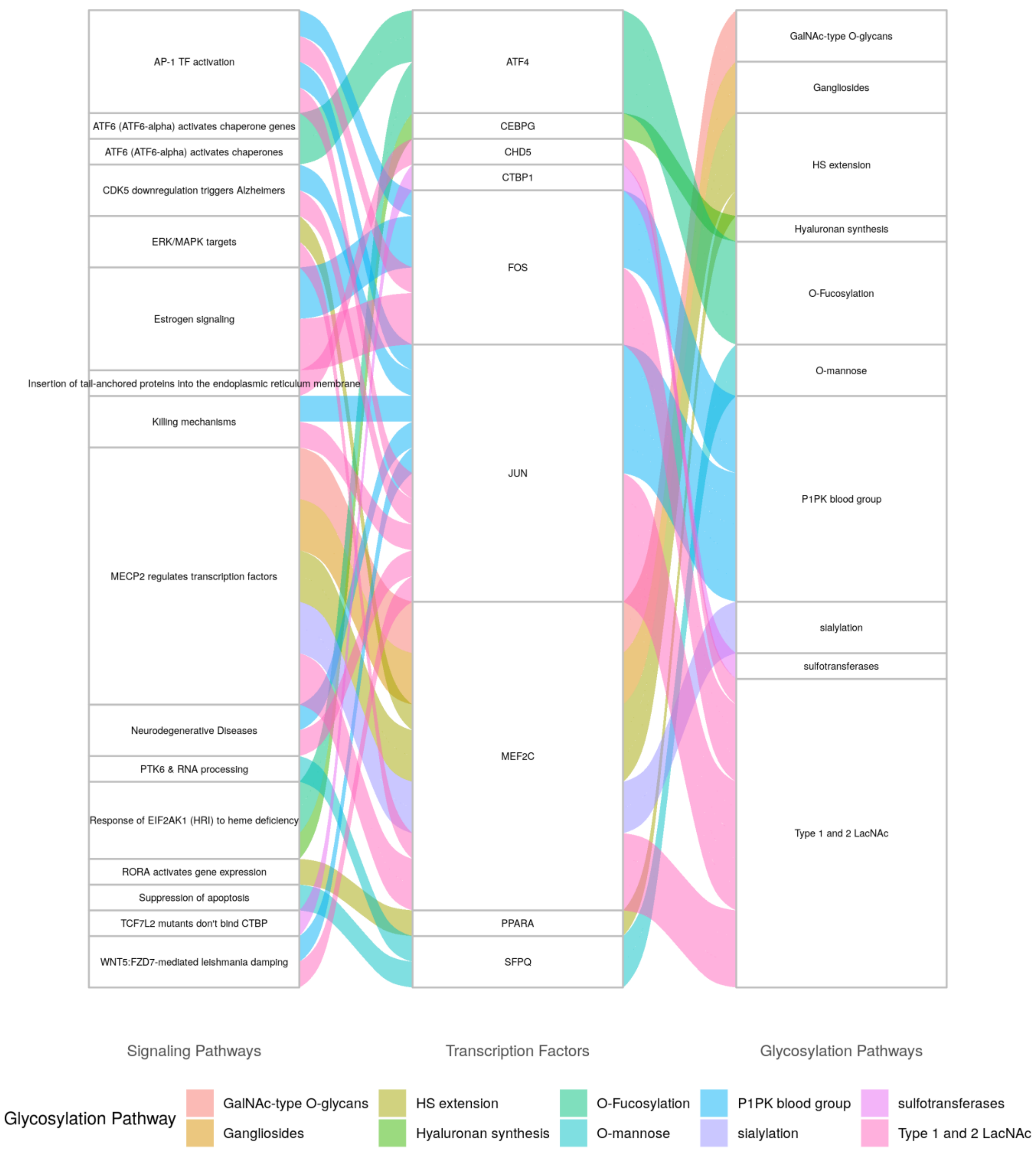

LIHC Signaling to Glycosylation Pathway Matching

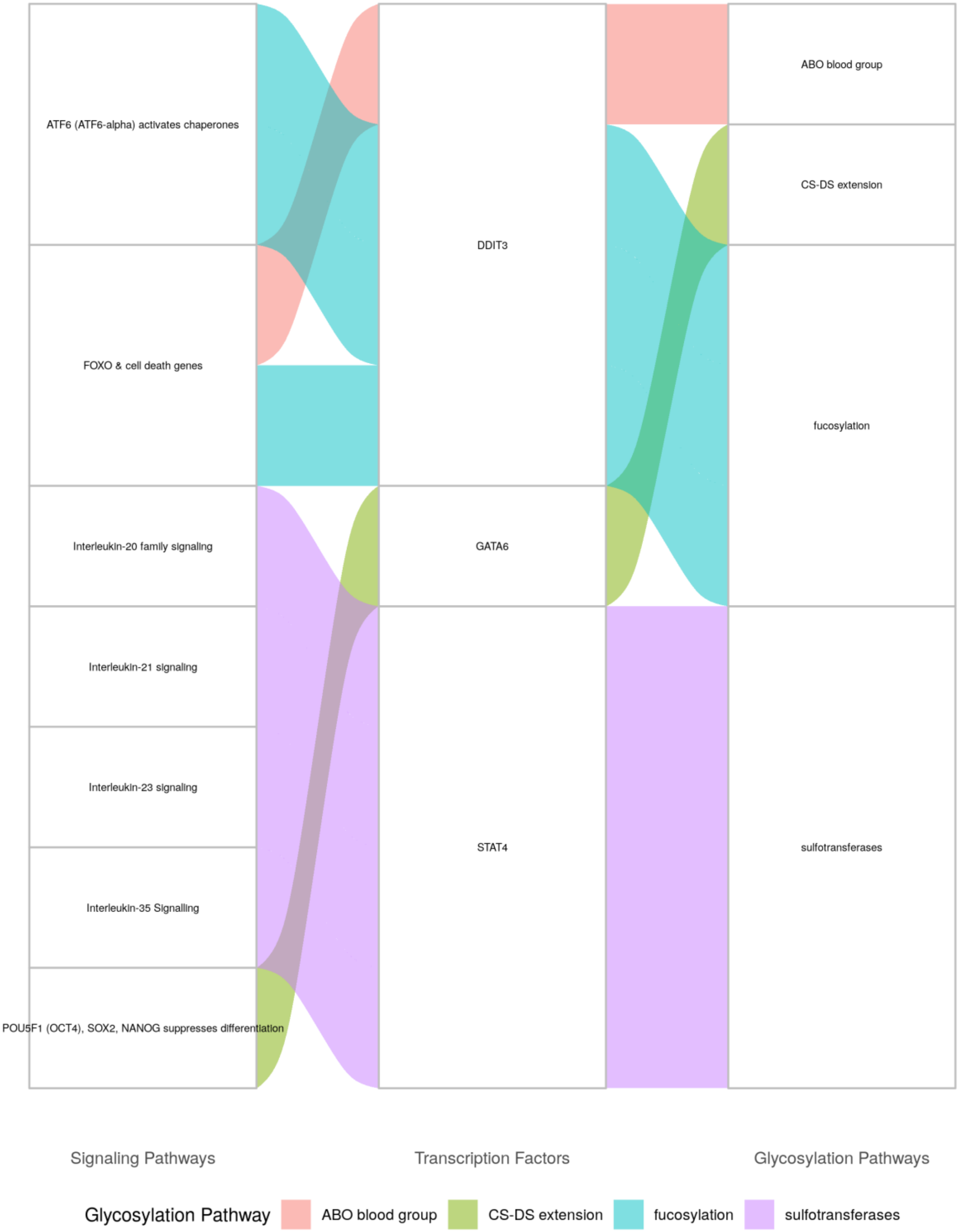

LUAD Signaling to Glycosylation Pathway Matching

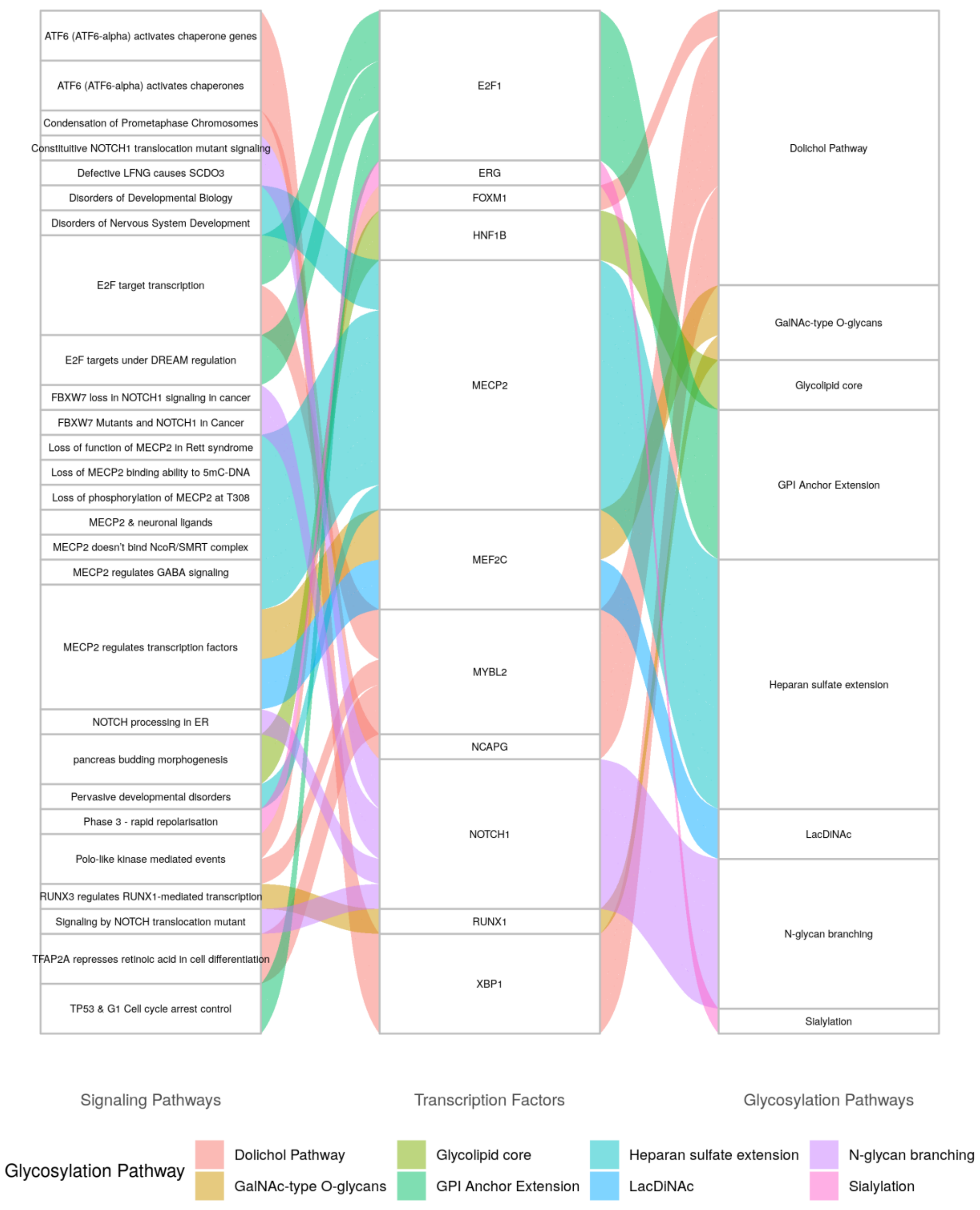

LUSC Signaling to Glycosylation Pathway Matching

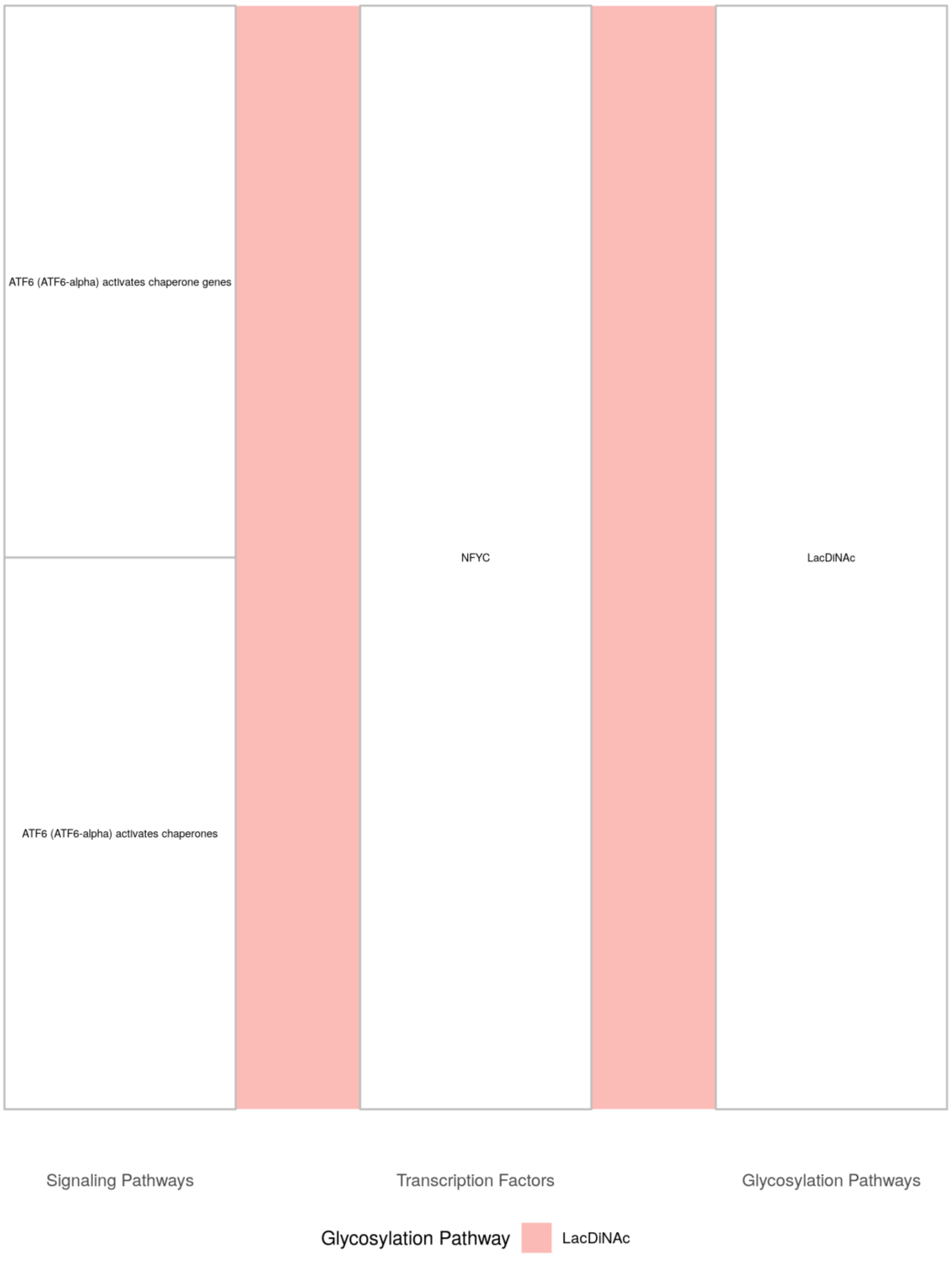

MESO Signaling to Glycosylation Pathway Matching

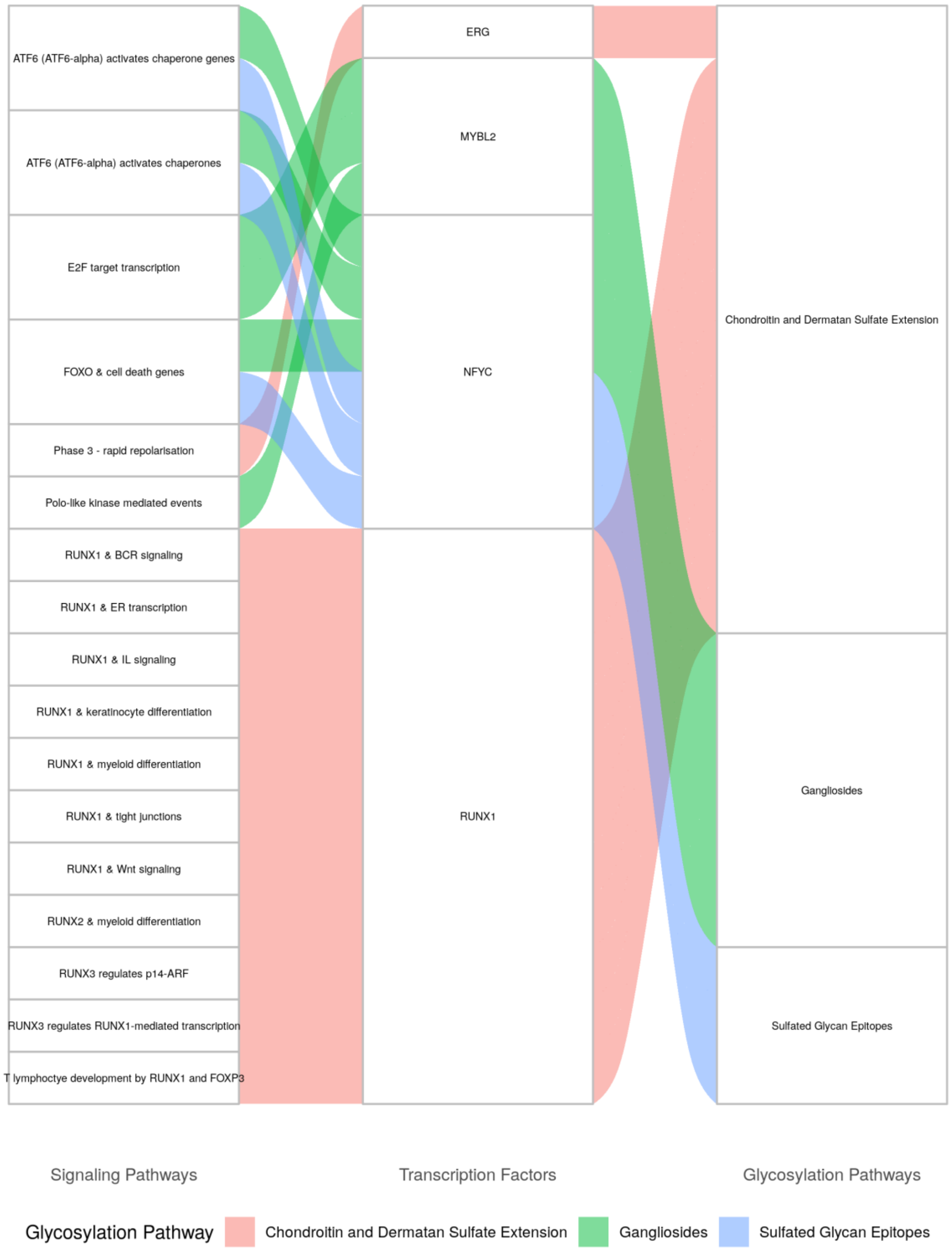

OV Signaling to Glycosylation Pathway Matching

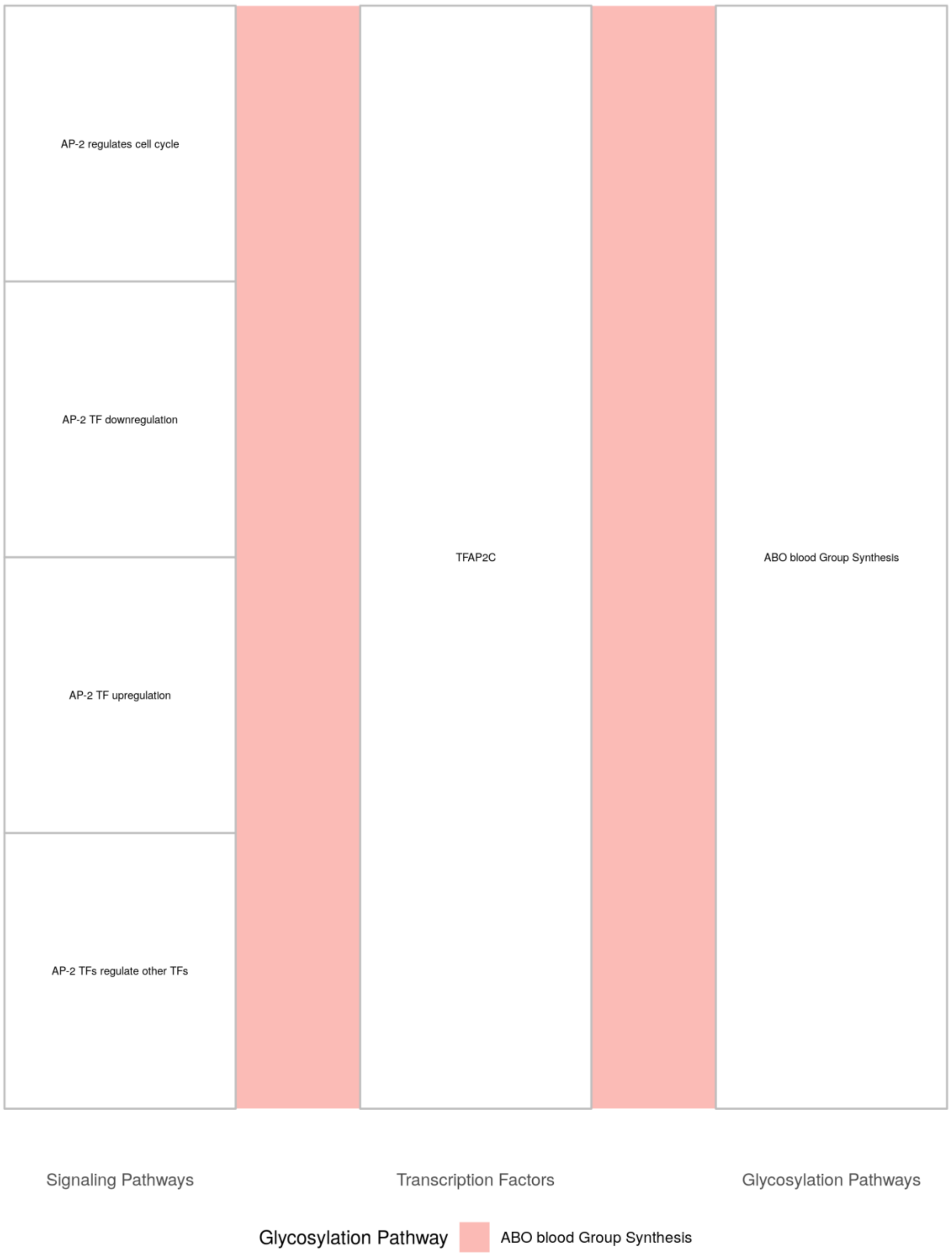

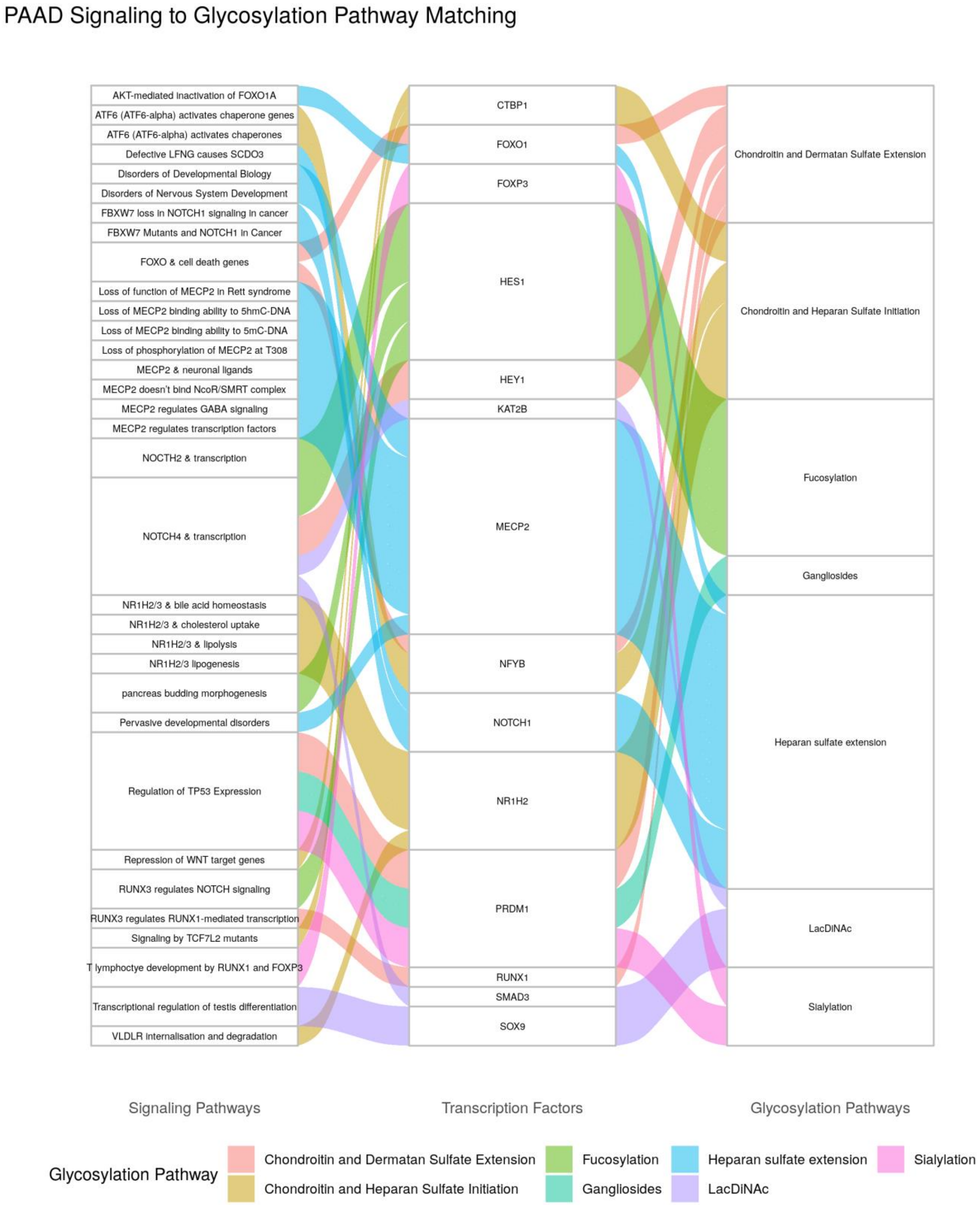
PAAD Signaling to Glycosylation Pathway Matching
AKT-mediated inactivation of FOXO1A
ATF6 (ATF6-alpha) activates chaperone genes
ATF6 (ATF6-alpha) activates chaperones
Defective LFNG causes SCDO3
Disorders of Developmental Biology
Disorders of Nervous System Development
FBXW7 loss in NOTCH1 signaling in cancer
FBXW7 Mutants and NOTCH1 in Cancer
FOXO & cell death genes
Loss of function of MECP2 in Rett syndrome
Loss of MECP2 binding ability to 5hmC-DNA
Loss of MECP2 binding ability to 5mC-DNA
Loss of phosphorylation of MECP2 at T308
MECP2 & neuronal ligands
MECP2 doesn't bind NcoR/SMRT complex
MECP2 regulates GABA signaling
MECP2 regulates transcription factors
NOCTH2 & transcription
NOTCH4 & transcription
NR1H2/3 & bile acid homeostasis
NR1H2/3 & cholesterol uptake
NR1H2/3 & lipolysis
NR1H2/3 lipogenesis
pancreas budding morphogenesis
Pervasive developmental disorders
Regulation of TP53 Expression
Repression of WNT target genes
RUNX3 regulates NOTCH signaling
RUNX3 regulates RUNX1-mediated transcription
Signaling by TCF7L2 mutants
T lymphoctye development by RUNX1 and FOXP3
Transcriptional regulation of testis differentiation
VLDLR internalisation and degradation
CTBP1
FOXO1
FOXP3
HES1
HEY1
KAT2B
MECP2
NFYB
NOTCH1
NR1H2
PRDM1
RUNX1
SMAD3
SOX9
Chondroitin and Dermatan Sulfate Extension
Chondroitin and Heparan Sulfate Initiation
Fucosylation
Gangliosides
Heparan sulfate extension
LacDiNAc
Sialylation
Signaling Pathways
Transcription Factors
Glycosylation Pathways
Glycosylation Pathway
Chondroitin and Dermatan Sulfate Extension
Chondroitin and Heparan Sulfate Initiation
Fucosylation
Gangliosides
Heparan sulfate extension
LacDiNAc
Sialylation

PCPG Signaling to Glycosylation Pathway Matching

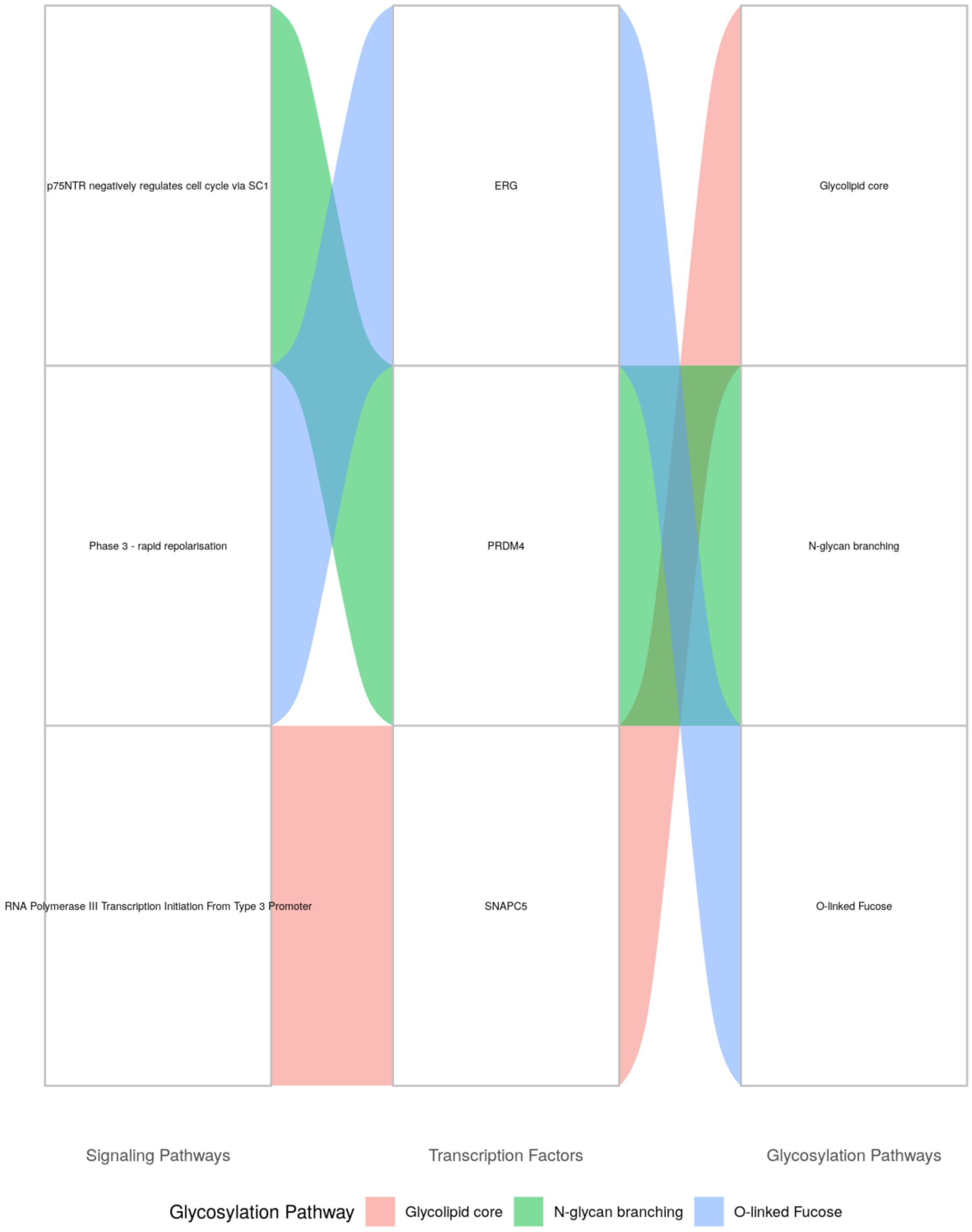

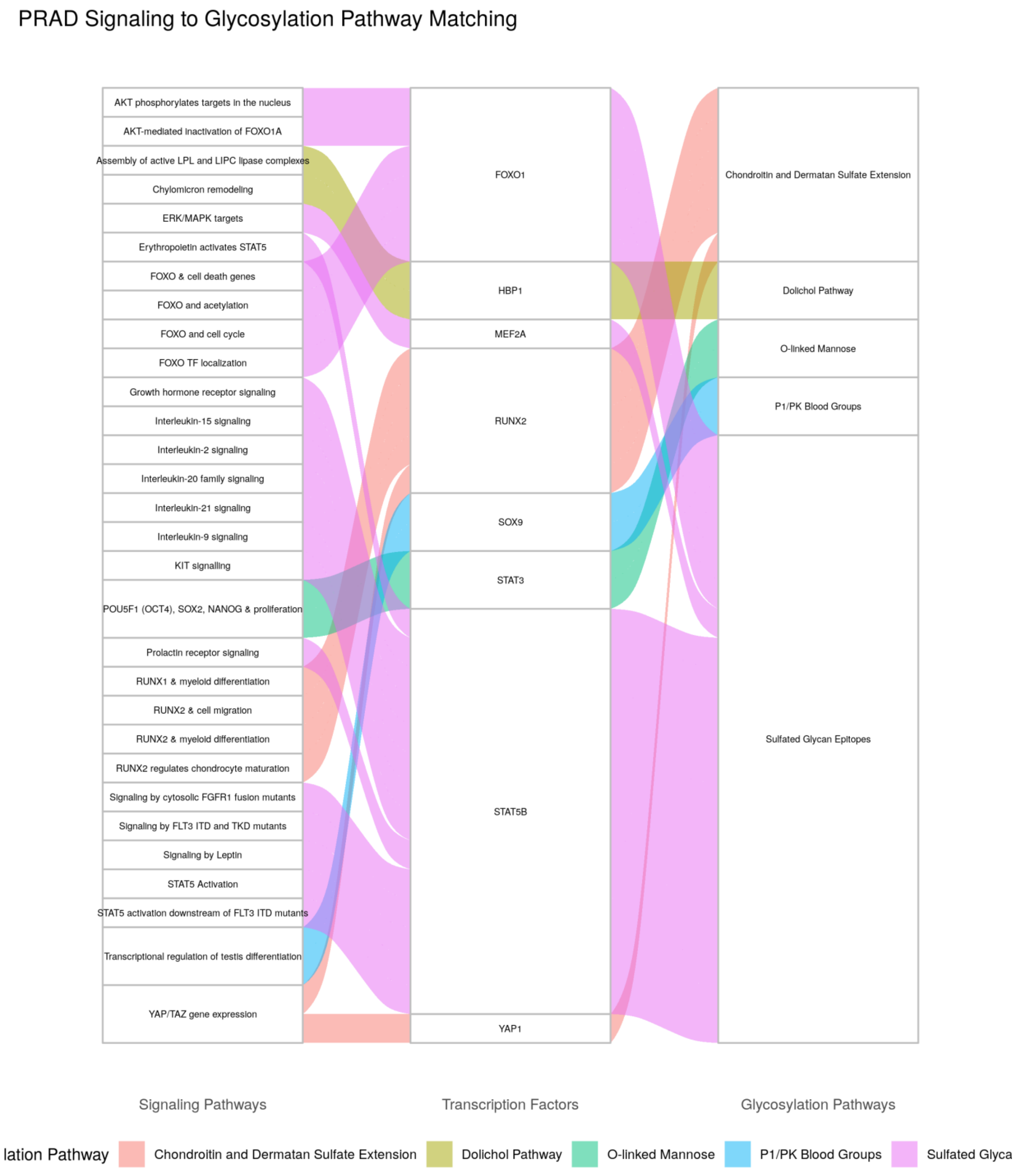

PRAD Signaling to Glycosylation Pathway Matching
AKT phosphorylates targets in the nucleus
AKT-mediated inactivation of FOXO1A
Assembly of active LPL and LIPC lipase complexes
Chylomicron remodeling
ERK/MAPK targets
Erythropoietin activates STAT5
FOXO & cell death genes
FOXO and acetylation
FOXO and cell cycle
FOXO TF localization
Growth hormone receptor signaling
Interleukin-15 signaling
Interleukin-2 signaling
Interleukin-20 family signaling
Interleukin-21 signaling
Interleukin-9 signaling
KIT signalling
POU5F1 (OCT4), SOX2, NANOG & proliferation
Prolactin receptor signaling
RUNX1 & myeloid differentiation
RUNX2 & cell migration
RUNX2 & myeloid differentiation
RUNX2 regulates chondrocyte maturation
Signaling by cytosolic FGFR1 fusion mutants
Signaling by FLT3 ITD and TKD mutants
Signaling by Leptin
STAT5 Activation
STAT5 activation downstream of FLT3 ITD mutants
Transcriptional regulation of testis differentiation
YAP/TAZ gene expression
FOXO1
HBP1
MEF2A
RUNX2
SOX9
STAT3
STAT5B
YAP1
Chondroitin and Dermatan Sulfate Extension
Dolichol Pathway
O-linked Mannose
P1/PK Blood Groups
Sulfated Glycan Epitopes
Signaling Pathways
Transcription Factors
Glycosylation Pathways
lation Pathway
Chondroitin and Dermatan Sulfate Extension
Dolichol Pathway
O-linked Mannose
P1/PK Blood Groups
Sulfated Glyca

SARC Signaling to Glycosylation Pathway Matching

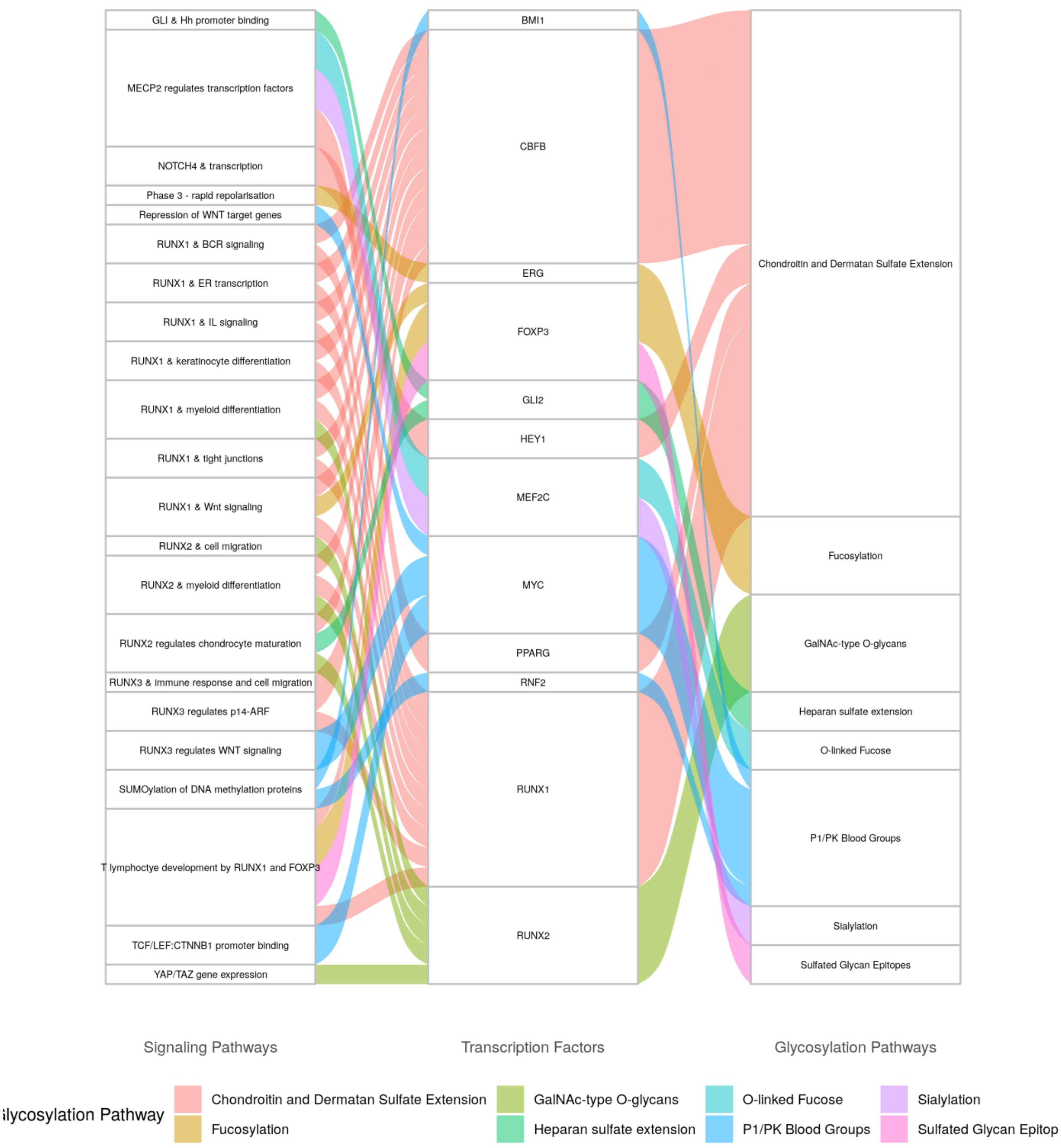

SKCM Signaling to Glycosylation Pathway Matching

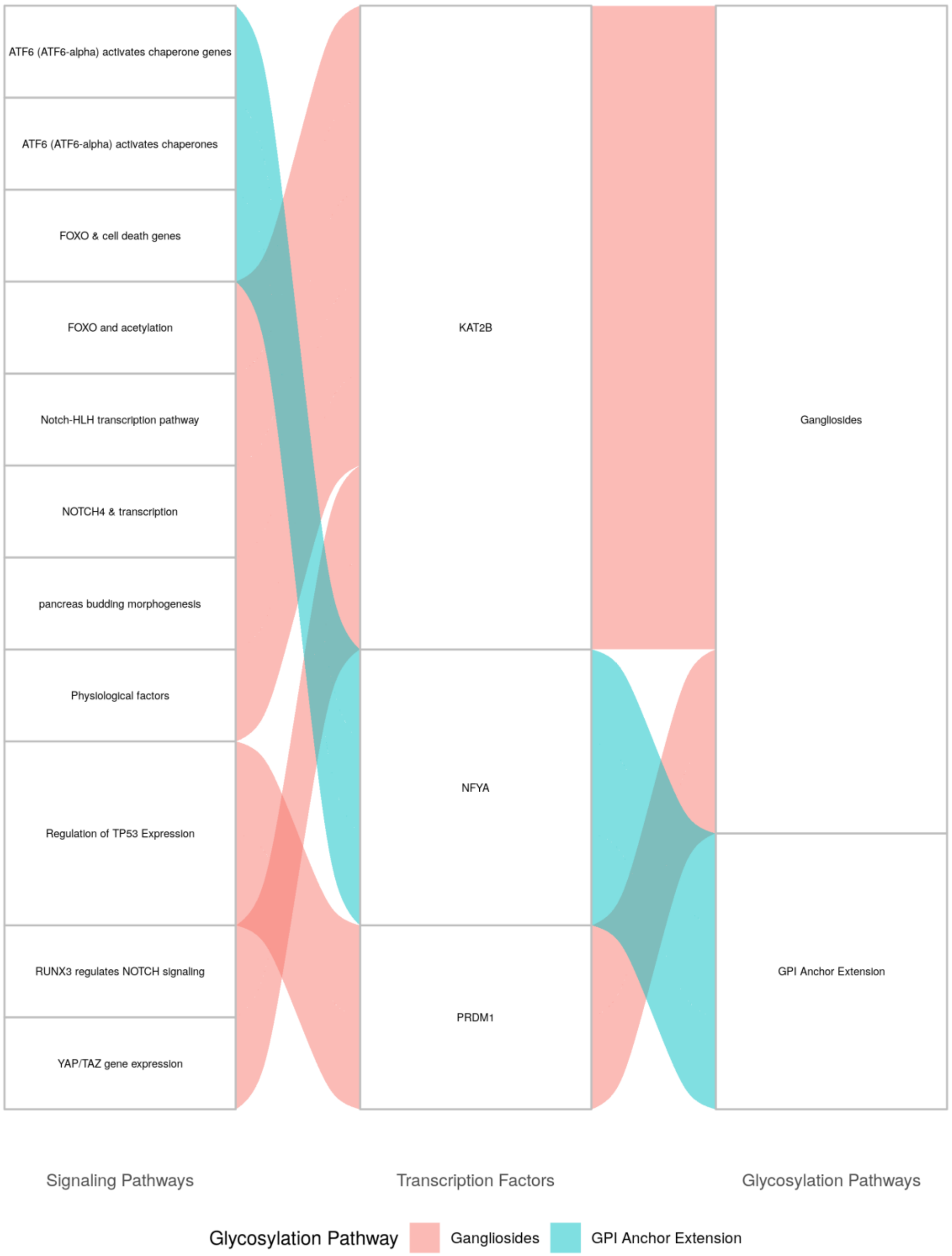

STES. Signaling to Glycosylation Pathway Matching

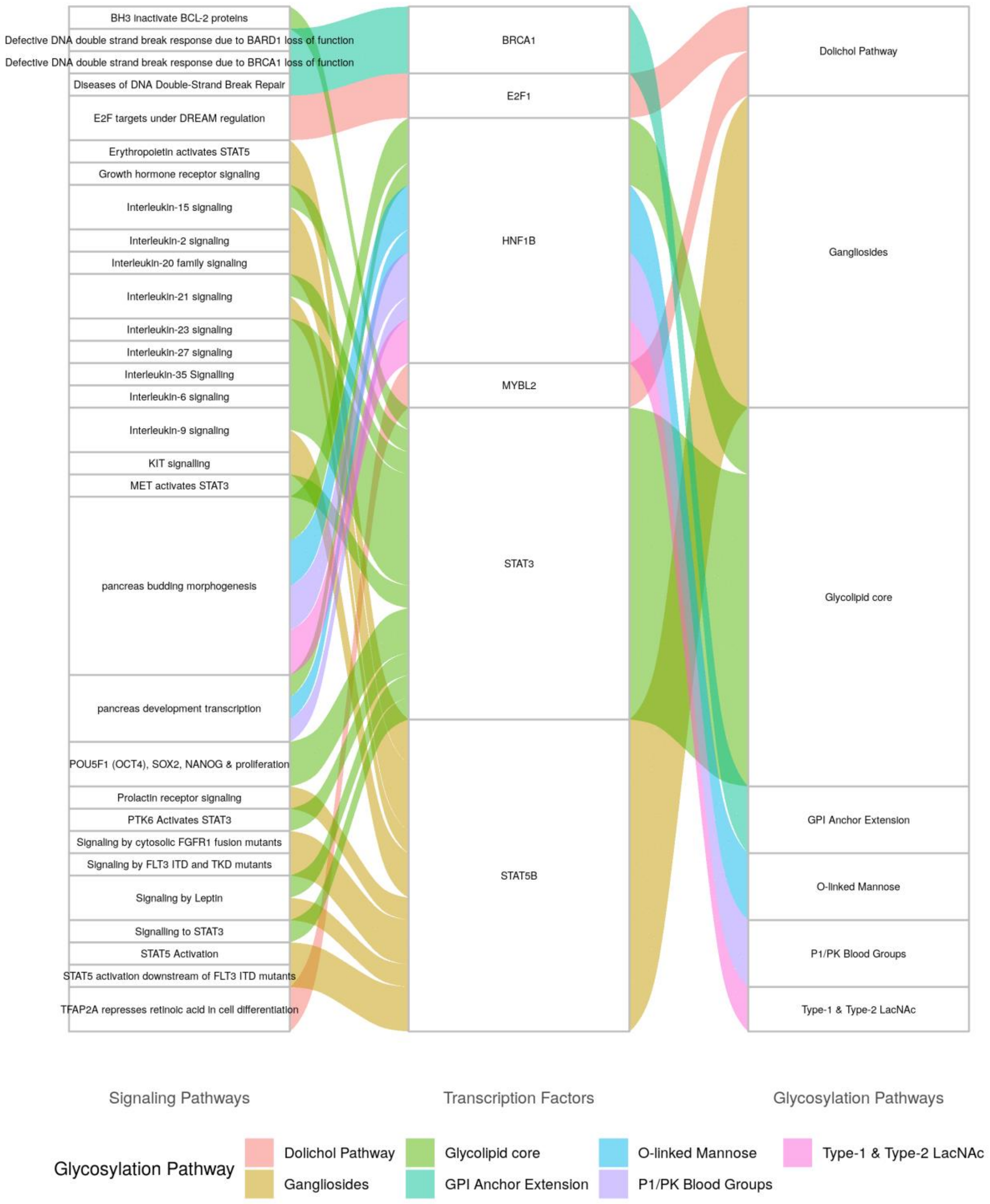

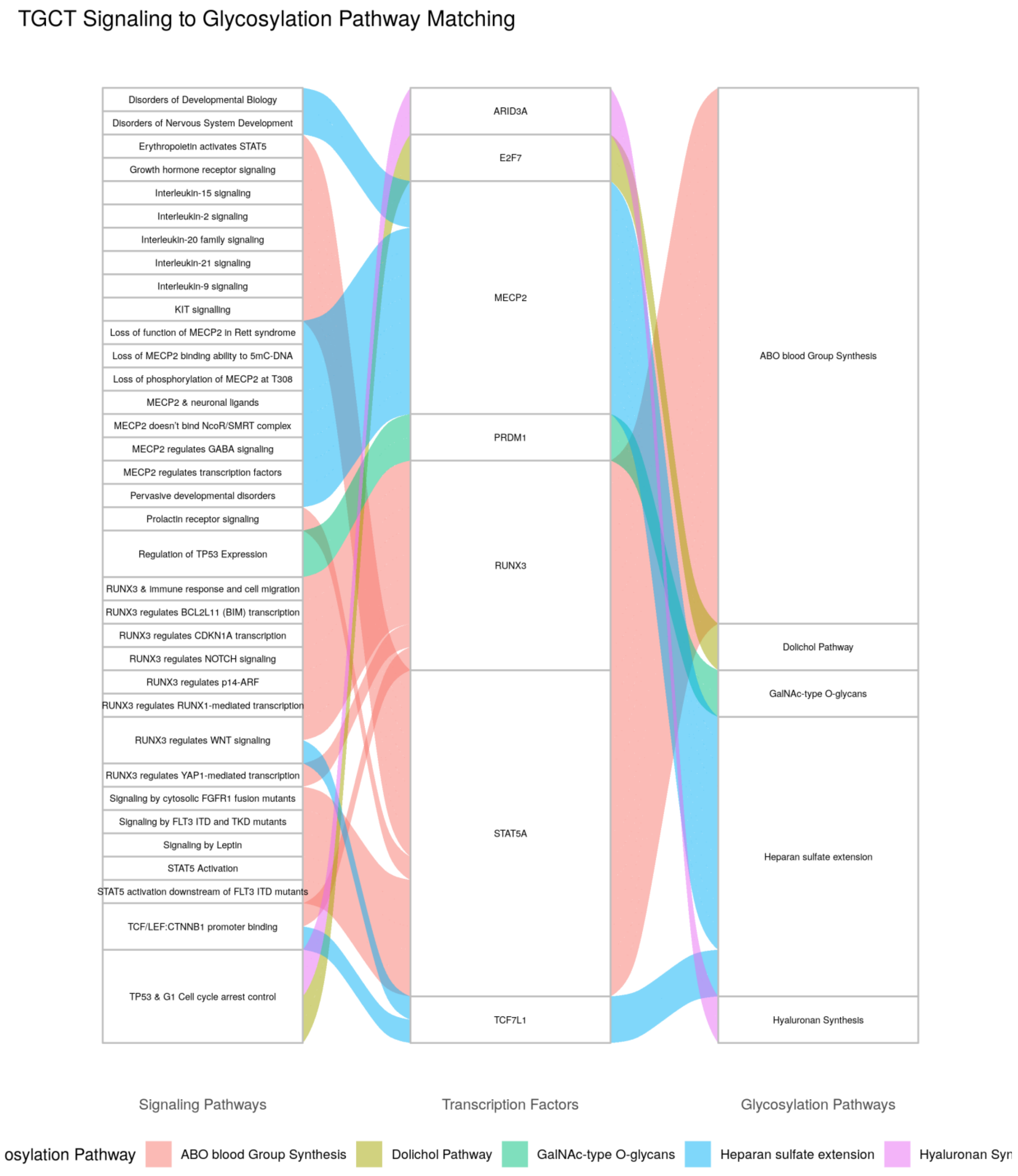
TGCT Signaling to Glycosylation Pathway Matching
Disorders of Developmental Biology
Disorders of Nervous System Development
Erythropoietin activates STAT5
Growth hormone receptor signaling
Interleukin-15 signaling
Interleukin-2 signaling
Interleukin-20 family signaling
Interleukin-21 signaling
Interleukin-9 signaling
KIT signalling
Loss of function of MECP2 in Rett syndrome
Loss of MECP2 binding ability to 5mC-DNA
Loss of phosphorylation of MECP2 at T308
MECP2 & neuronal ligands
MECP2 doesn't bind NcoR/SMRT complex
MECP2 regulates GABA signaling
MECP2 regulates transcription factors
Pervasive developmental disorders
Prolactin receptor signaling
Regulation of TP53 Expression
RUNX3 & immune response and cell migration
RUNX3 regulates BCL2L11 (BIM) transcription
RUNX3 regulates CDKN1A transcription
RUNX3 regulates NOTCH signaling
RUNX3 regulates p14-ARF
RUNX3 regulates RUNX1-mediated transcription
RUNX3 regulates WNT signaling
RUNX3 regulates YAP1-mediated transcription
Signaling by cytosolic FGFR1 fusion mutants
Signaling by FLT3 ITD and TKD mutants
Signaling by Leptin
STAT5 Activation
STAT5 activation downstream of FLT3 ITD mutants
TCF/LEF:CTNNB1 promoter binding
TP53 & G1 Cell cycle arrest control
ARID3A
E2F7
MECP2
PRDM1
RUNX3
STAT5A
TCF7L1
ABO blood Group Synthesis
Dolichol Pathway
GalNAc-type O-glycans
Heparan sulfate extension
Hyaluronan Synthesis
Signaling Pathways
Transcription Factors
Glycosylation Pathways
osylation Pathway
ABO blood Group Synthesis
Dolichol Pathway
GalNAc-type O-glycans
Heparan sulfate extension
Hyaluronan Syn

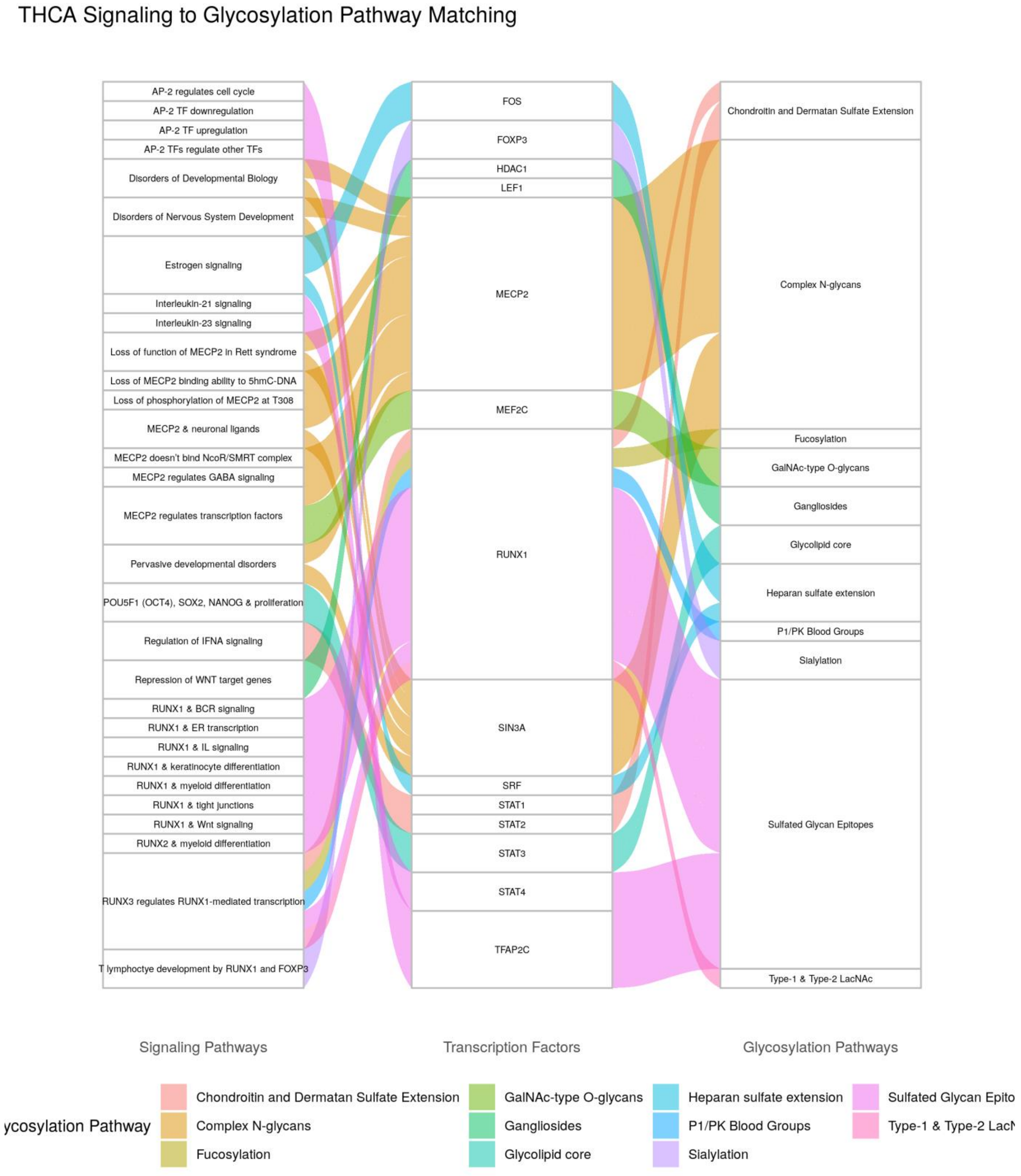
THCA Signaling to Glycosylation Pathway Matching
AP-2 regulates cell cycle
AP-2 TF downregulation
AP-2 TF upregulation
AP-2 TFs regulate other TFs
Disorders of Developmental Biology
Disorders of Nervous System Development
Estrogen signaling
Interleukin-21 signaling
Interleukin-23 signaling
Loss of function of MECP2 in Rett syndrome
Loss of MECP2 binding ability to 5hmC-DNA
Loss of phosphorylation of MECP2 at T308
MECP2 & neuronal ligands
MECP2 doesn't bind NcoR/SMRT complex
MECP2 regulates GABA signaling
MECP2 regulates transcription factors
Pervasive developmental disorders
POU5F1 (OCT4), SOX2, NANOG & proliferation
Regulation of IFNA signaling
Repression of WNT target genes
RUNX1 & BCR signaling
RUNX1 & ER transcription
RUNX1 & IL signaling
RUNX1 & keratinocyte differentiation
RUNX1 & myeloid differentiation
RUNX1 & tight junctions
RUNX1 & Wnt signaling
RUNX2 & myeloid differentiation
RUNX3 regulates RUNX1-mediated transcription
T lymphoctye development by RUNX1 and FOXP3
FOS
FOXP3
HDAC1
LEF1
MECP2
MEF2C
RUNX1
SIN3A
SRF
STAT1
STAT2
STAT3
STAT4
TFAP2C
Chondroitin and Dermatan Sulfate Extension
Complex N-glycans
Fucosylation
GalNAc-type O-glycans
Gangliosides
Glycolipid core
Heparan sulfate extension
P1/PK Blood Groups
Sialylation
Sulfated Glycan Epitopes
Type-1 & Type-2 LacNAc
Signaling Pathways
Transcription Factors
Glycosylation Pathways
ycosylation Pathway
Chondroitin and Dermatan Sulfate Extension
Complex N-glycans
Fucosylation
GalNAc-type O-glycans
Gangliosides
Glycolipid core
Heparan sulfate extension
P1/PK Blood Groups
Sialylation
Sulfated Glycan Epito
Type-1 & Type-2 LacN

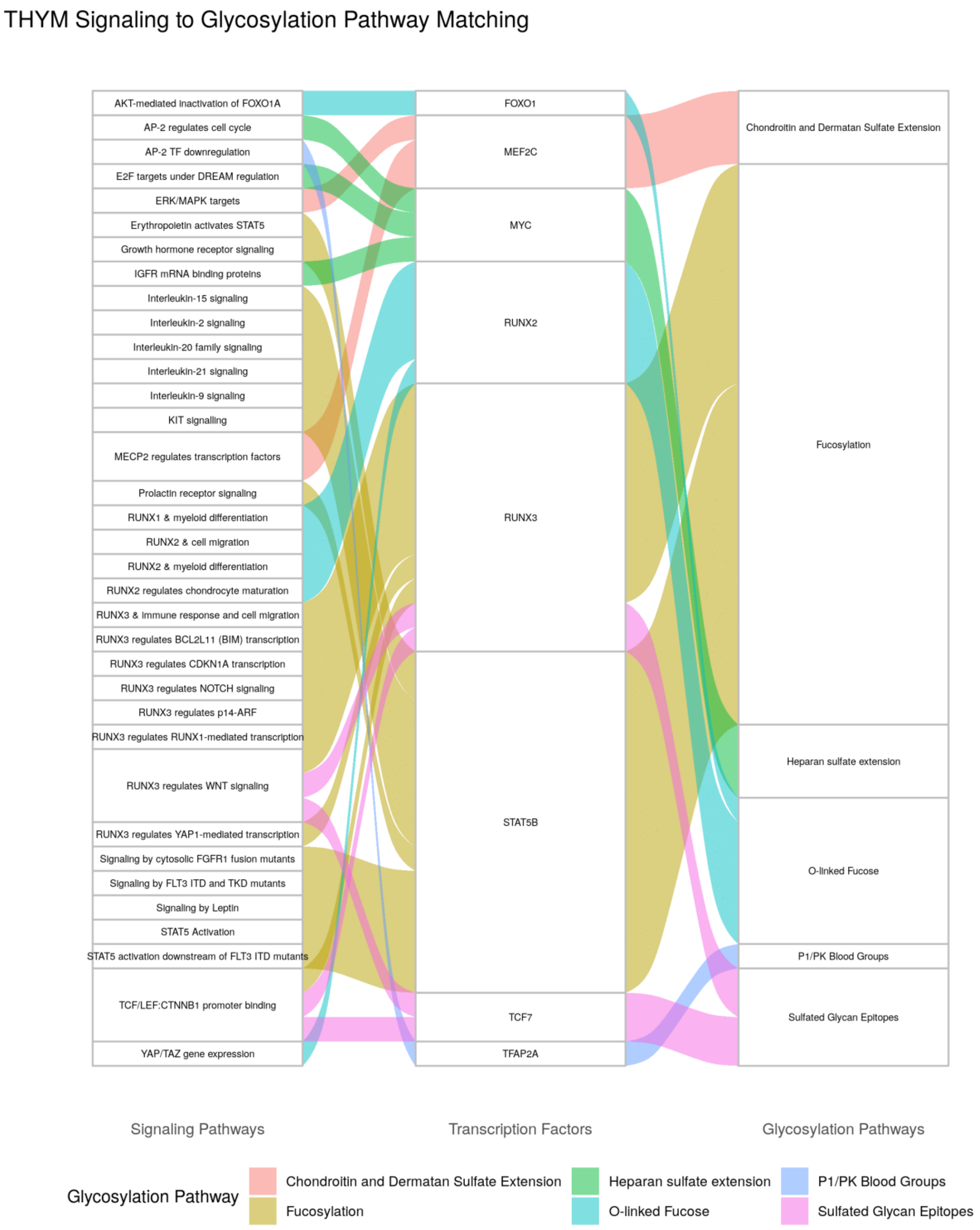
THYM Signaling to Glycosylation Pathway Matching
AKT-mediated inactivation of FOXO1A
AP-2 regulates cell cycle
AP-2 TF downregulation
E2F targets under DREAM regulation
ERK/MAPK targets
Erythropoietin activates STAT5
Growth hormone receptor signaling
IGFR mRNA binding proteins
Interleukin-15 signaling
Interleukin-2 signaling
Interleukin-20 family signaling
Interleukin-21 signaling
Interleukin-9 signaling
KIT signalling
MECP2 regulates transcription factors
Prolactin receptor signaling
RUNX1 & myeloid differentiation
RUNX2 & cell migration
RUNX2 & myeloid differentiation
RUNX2 regulates chondrocyte maturation
RUNX3 & immune response and cell migration
RUNX3 regulates BCL2L11 (BIM) transcription
RUNX3 regulates CDKN1A transcription
RUNX3 regulates NOTCH signaling
RUNX3 regulates p14-ARF
RUNX3 regulates RUNX1-mediated transcription
RUNX3 regulates WNT signaling
RUNX3 regulates YAP1-mediated transcription
Signaling by cytosolic FGFR1 fusion mutants
Signaling by FLT3 ITD and TKD mutants
Signaling by Leptin
STAT5 Activation
STAT5 activation downstream of FLT3 ITD mutants
TCF/LEF:CTNNB1 promoter binding
YAP/TAZ gene expression
FOXO1
MEF2C
MYC
RUNX2
RUNX3
STAT5B
TCF7
TFAP2A
Chondroitin and Dermatan Sulfate Extension
Fucosylation
Heparan sulfate extension
O-linked Fucose
P1/PK Blood Groups
Sulfated Glycan Epitopes
Signaling Pathways
Transcription Factors
Glycosylation Pathways
Glycosylation Pathway
Chondroitin and Dermatan Sulfate Extension
Fucosylation
Heparan sulfate extension
O-linked Fucose
P1/PK Blood Groups
Sulfated Glycan Epitopes

UCEC Signaling to Glycosylation Pathway Matching

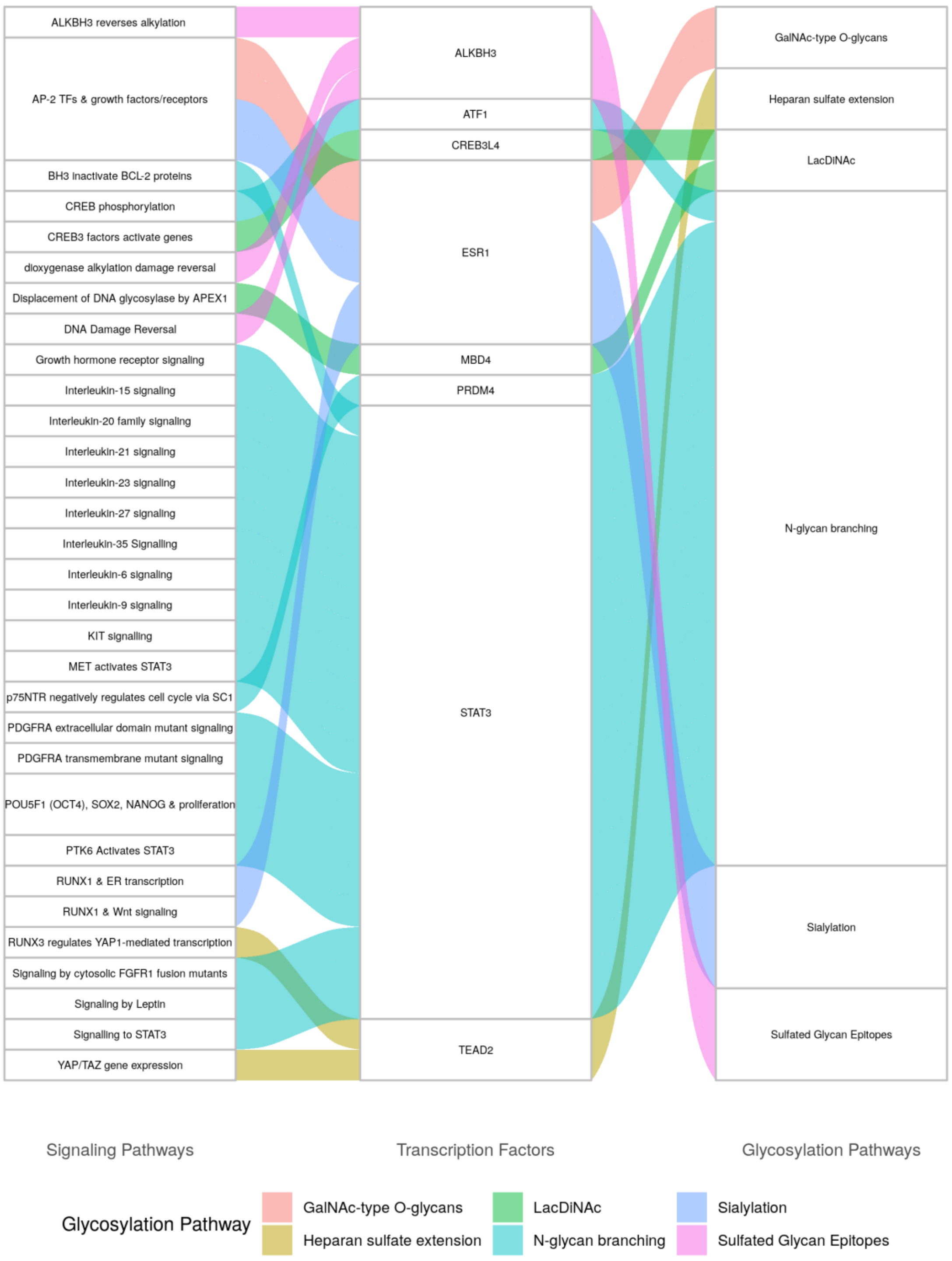

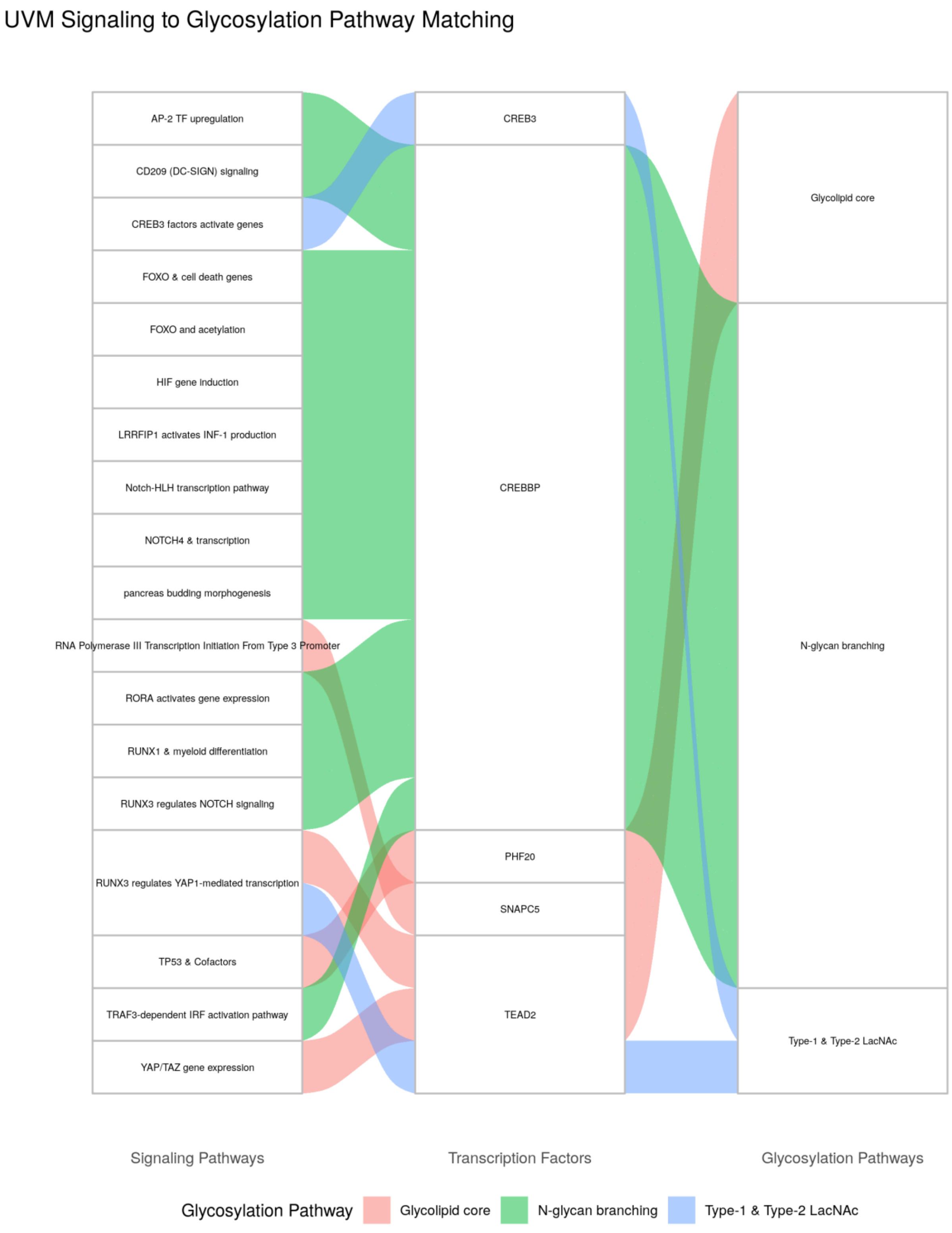
UVM Signaling to Glycosylation Pathway Matching
AP-2 TF upregulation
CD209 (DC-SIGN) signaling
CREB3 factors activate genes
FOXO & cell death genes
FOXO and acetylation
HIF gene induction
LRRFIP1 activates INF-1 production
Notch-HLH transcription pathway
NOTCH4 & transcription
pancreas budding morphogenesis
RNA Polymerase III Transcription Initiation From Type 3 Promoter
RORA activates gene expression
RUNX1 & myeloid differentiation
RUNX3 regulates NOTCH signaling
RUNX3 regulates YAP1-mediated transcription
TP53 & Cofactors
TRAF3-dependent IRF activation pathway
YAP/TAZ gene expression
CREB3
CREBBP
PHF20
SNAPC5
TEAD2
Glycolipid core
N-glycan branching
Type-1 & Type-2 LacNAc
Signaling Pathways
Transcription Factors
Glycosylation Pathways
Glycosylation Pathway
Glycolipid core
N-glycan branching
Type-1 & Type-2 LacNAc
